# Supplementary material for: Sex-specificity of the C. elegans metabolome
Source: Nat Commun. 2023 Jan 19;14:320. doi: 10.1038/s41467-023-36040-y (PMC9852247; doi:10.1038/s41467-023-36040-y)
Supplement: Supplementary file 1 — Supplementary Information [file 41467_2023_36040_MOESM1_ESM.pdf]

Supplementary Information

Sex-specificity of the *C. elegans* metabolome

Russell N. Burkhardt[1], Alexander B. Artyukhin[1,2], Erin Z. Aprison[3], Brian J. Curtis[1], Bennett W. Fox[1], Andreas H. Ludewig[1], Diana Fajardo Palomino[1], Jintao Luo[4,5], Amaresh Chaturbedi[6], Oishika Panda[1], Chester J. J. Wrobel[1], Victor Baumann[1], Douglas S. Portman[4], Siu S. Lee[6], Ilya Ruvinsky[3,*], and Frank C. Schroeder[1,*]

[1]Boyce Thompson Institute and Department of Chemistry and Chemical Biology, Cornell University, Ithaca, New York 14853, United States

[2]Current address: Chemistry Department, College of Environmental Science and Forestry, State University of New York, Syracuse, New York 13210, United States

[3]Department of Molecular Biosciences, Northwestern University, Evanston, IL 60208, United States

[4]Department of Biomedical Genetics, University of Rochester, Rochester, NY, 14642, United States.

[5]Current address: School of Life Sciences, Xiamen University, Xiamen, Fujian, 361102, China.

[6]Department of Molecular Biology and Genetics, Cornell University, Ithaca, New York 14853, United States

*Correspondence to fs31@cornell.edu and ilya.ruvinsky@northwestern.edu

Contents

1. Chemical Synthesis 2

2. Supplementary Figures 6

3. NMR Spectra 33

4. Supplementary References 43

1. Chemical Synthesis

Chemicals and General Synthetic Procedures. Unless stated otherwise, all chemicals purchased for use were obtained from MilliporeSigma (Burlington, MA). *L*-isoleucine *tert*-butyl ester hydrochloride, trifluoroacetic acid, and *N*,*N'*-dicyclohexylcarbodiimide (DCC) were obtained from TCI (Portland, OR), 4-dimethylaminopyridine was purchased from Fluka, and L-tryptophan *tert*-butyl ester hydrochloride was purchased from Chem-Impex.. 1-hydroxybenzotriazole (HOBt) was obtained from Fluka (Mexico City, Mexico). All deuterated solvents were obtained from Cambridge Isotopes. All non-deuterated solvents were obtained from Fisher Sci (Waltham, MA). Abbreviations used for solvents are as follows: chloroform ($CHCl_3$), dichloromethane (DCM), dimethylsulfoxide (DMSO), and methanol (MeOH).

Unless stated otherwise, all reactions were performed under argon atmosphere in flame-dried glassware. All commercially available reagents were used as purchased unless otherwise stated. All solvents were dried over activated 3Å sieves for a minimum of 24 h unless used in reactions where aqueous reagents were involved. Thin-layer chromatography (TLC) was performed with J.T. Baker Silica Gel IB2-F plastic-backed plates. Reversed-phase column chromatography was performed using Teledyne ISCO CombiFlash Rf and Rf+ systems with Teledyne ISCO RediSep Rf and Rf Gold silica columns. Nuclear Magnetic Resonance (NMR) spectra were recorded on a Varian INOVA 600 (600 MHz) or Bruker AV 500 (500 MHz) in the Cornell University NMR Facility. ^{1}H NMR chemical shifts are reported in ppm (δ) relative to the residual solvent peaks (7.26 ppm for $CDCl_3$, 3.31 ppm for CD_3OD, and 2.50 for D_6-DMSO) and ^{13}C NMR shifts relative to the residual solvent peaks (77.16 for $CDCl_3$ and 49.00 for CD_3OD, and 39.52 for D_6-DMSO). All NMR data processing was done using MNOVA 14.2.3 (https://mestrelab.com/).

1.1. Synthesis of acemta#1 and acemta#2

***(2O)-* and *(3O)*-acetyl-*S*-methylthioadenosine (acemta#1, 4, and acemta#2, 5)**

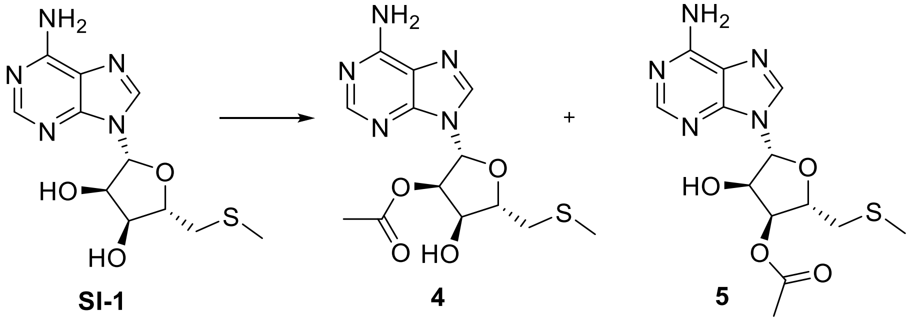

SI-1 **4** **5**

S-Methylthioadenosine (MTA, **SI-1**, 10 mg, 0.0336 mmol) was dissolved in pyridine (150 µL) and stirred at ambient temperature. Acetic anhydride (34 µL, 0.0336 mmol) was added and the reaction stirred. After 2 h (MeOH, 2 mL) was added and the reaction stirred 30 min. The reaction was diluted with $CHCl_3$ and washed with 2% aqueous acetic acid (1×4 mL) and saturated sodium bicarbonate (1×6 mL) then dried over magnesium sulfate ($MgSO_4$), filtered, and concentrated under reduced pressure. The resulting oil was purified by flash chromatography on silica. Elution with a gradient of 0-40% DCM/MeOH yielded the diacetylated product (10 mg, 74.9%) and a 1:3 mixture of the (2*O*)- and (3*O*)-acetylated products (acemta#1, **4**, and acemta#2, **5**, 2.5 mg, 20.9%) as an oil.

***(2O)*-acetyl *S*-methylthioadenosine (4, acemta#1):**

^{1}H NMR ($CDCl_3$, 500 MHz): δ (ppm) 8.31 (s, 1H), 7.98 (s, 1H), 6.09 (d, *J* = 3.4 Hz, 1H), 5.75 (dd, *J* = 5.6, 3.4 Hz, 1H), 4.83 (t, *J* = 6.3 Hz, 1H), 4.25 (q, *J* = 6.0 Hz, 1H), 2.99 (dd, *J* = 14.2, 5.2 Hz, 1H), 1.85-2.95 (m, 1H), 2.17 (s, 3H), 2.15 (s, 3H).

^{13}C NMR ($CDCl_3$, 125 MHz): δ (ppm) 170.4, 155.5, 153.1, 149.5, 139.7, 120.0, 87.2, 83.2, 76.1, 72.2, 36.4, 20.9, 16.9.

HRMS (ESI) *m/z*: Calculated: $(M+H)^+$ 340.1074. Actual: 340.1049. Δ ppm: -7.30.

***(3O)*-acetyl *S*-methylthioadenosine (5, acemta#2):**

^{1}H NMR ($CDCl_3$, 500 MHz): δ (ppm) 8.25 (s, 1H), 8.02 (s, 1H), 5.94 (d, *J* = 6.7 Hz, 1H), 5.38 (dd, *J* = 5.8, 2.8 Hz, 1H), 4.97 (t, *J* = 6.3 Hz, 1H), 4.48 (td, *J* = 5.6, 2.8 Hz, 1H), 2.90 (t, *J* = 5.5 Hz, 2H), 2.21 (s, 3H), 2.16 (s, 3H).

^{13}C NMR ($CDCl_3$, 125 MHz): δ (ppm) 170.6, 155.6, 152.7 (2C), 139.3, 89.5, 83.5, 75.0, 73.8, 37.1, 21.1, 17.0.

1.2. Synthesis of medip#1

***N,N*-Dimethyltryptophan (24)**

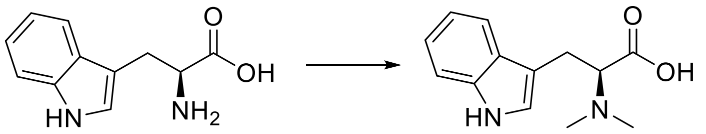

SI-2 **24**

Based on a synthesis for *N,N*-dimethyltryptamine[9], tryptophan (**SI-2**, 1.0 g, 4.9 mmol) was stirred in MeOH (30 mL) at 0 °C under an atmosphere of argon. Sodium cyanoborohydride (1.5 g, 24 mmol) was added followed by formaldehyde solution (1.54 mL, 36%, 20 mmol) and the reaction was stirred for 36 h. The reaction was concentrated and recrystallized from MeOH to yield **24** (636 mg, 56%) as white crystals that were used without further purification.

^{1}H NMR (D_6-DMSO, 500 MHz): δ (ppm) 10.83 (s, 1H), 7.54 (d, *J* = 7.8 Hz, 1H), 7.32 (d, *J* = 7.8 Hz, 1H), 7.06 (td, *J* = 7.2, 1.0 Hz, 1H), 6.97 (td, *J* = 7.2, 1.0 Hz, 1H), 3.47 (dd, *J* = 8.0, 6.1 Hz, 1H), 3.34 (dd, *J* = 14.8, 7.9 Hz, 1H), 2.97 (dd, *J* = 14.7, 6.2 Hz), 2.41 (s, 6H).

^{13}C NMR (D_6-DMSO, 125 MHz): δ (ppm) 171.0, 136.1, 127.2, 123.4, 120.9, 118.3, 118.2, 111.3, 110.5, 68.4, 41.3, 24.3.

HRMS (ESI) *m/z*: Calculated: $(M+H)^+$ 233.1289. Actual: 233.1270. Δ ppm: 4.27.

medip#1-*tert*-butyl ester (SI-3)

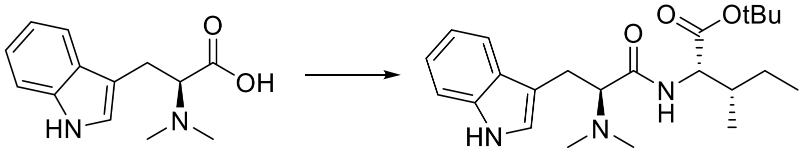

24 **SI-3**

HOBt (11 mg, 0.0647 mmol), triethylamine (15 μL, 0.108 mmol), DCC (11.6 mg, 0.056 mmol), and isoleucine-*tert*-butyl ester (20 mg, 0.086 mmol) were added to a solution of *N,N*-dimethyltryptophan (**24**, 10 mg, 0.043 mmol) in DCM (1 mL) and DMF (1 mL) and stirred overnight. The reaction was quenched with water (1 mL), extracted with DCM (3×5 mL), dried over $MgSO_4$, filtered, and concentrated under reduced pressure. The resulting solid was purified via flash chromatography on silica gel. Elution with a gradient of 0-10% DCM/MeOH yielded a mixture of medip#1-*tert*-butyl ester (**SI-3**) and isoleucine-*tert*-butyl ester (27.8 mg), which was used without further purification.

medip#1 (26)

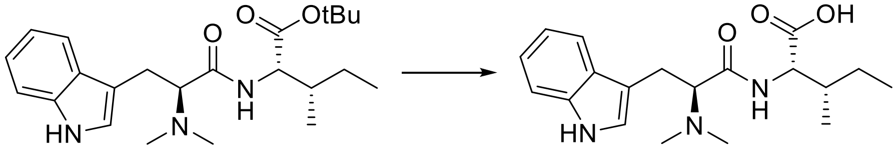

SI-3 **26**

TFA (530 μL, 6.88 mmol) was added to a stirring solution of medip#1-*tert*-butyl ester (**SI-3**) and isoleucine-*tert*-butyl ester (27.8 mg) in DCM (530 μL) and stirred for 4 h. The reaction was then concentrated under reduced pressure. The resulting oil was purified via flash chromatography on silica gel. Elution with a gradient of 0-20% DCM/MeOH yielded medip#1 (**26**) as a white solid (14.9 mg, ~100%).

^{1}H NMR (CD_3OD, 500 MHz): δ (ppm) 7.59 (d, *J* = 8.0 Hz, 1H), 7.36 (d, *J* = 8.1 Hz, 1H), 7.22 (s, 1H), 7.12 (td, *J* = 7.3, 1 Hz, 1H), 7.05 (td, *J* = 7.3, 1 Hz, 1H), 4.29 (d, *J* = 5.5 Hz, 1H), 4.22 (dd, *J* = 8.9, 5.3 Hz, 1H), 3.50 (dd, *J* = 14.6, 5.2 Hz, 1H), 3.42 (dd, *J* = 14.6, 8.9 Hz, 1H), 2.98 (s, 6H), 1.78-1.89 (m, 1H), 1.35-1.48 (m, 1H), 1.08-1.18 (m, 1H), 0.89 (t, *J* = 7.5 Hz, 3H), 0.88 (d, *J* = 6.8 Hz, 3H).

^{13}C NMR (CD_3OD, 125 MHz): δ (ppm) 173.2, 168.5, 138.1, 128.2, 125.6, 120.3, 118.8, 112.6, 107.2, 69.5, 58.4, 54.8, 42.4, 38.4, 26.2, 25.7, 15.9, 11.7.

HRMS (ESI) *m/z*: Calculated: $(M+H)^+$ 346.2125. Actual: 346.2102. Δ ppm: 6.77.

1.3. Synthesis of Trp-Ile and Ile-Trp

L-Tryptophanyl-L-isoleucine (SI-5)

To a vial containing N-(*tert*-butoxycarbonyl)-L-tryptophan (50 mg, 0.16 mmol, 1.0 equiv.), L-isoleucine *tert*-butyl ester hydrochloride (48 mg, 0.0.21 mmol, 1.3 equiv.), 1-ethyl-3-(3′-dimethylaminopropyl)carbodiimide (EDC) hydrochloride (108 mg, 0.56 mmol, 3.4 equiv.) and 4-dimethylaminopyridine (DMAP, 40 mg, 0.33 mmol, 2.0 equiv.) was added about 1 mL of DCM. The resulting solution was stirred at room temperature overnight and concentrated in vacuo. Flash column chromatography on silica using a gradient of 2-54% ethyl acetate in n-hexane afforded [*N*-(*tert*-butoxycarbonyl)-L-tryptophanyl]-L-isoleucine *tert*-butyl ester (**SI-4**) (73 mg, 94%).

To a solution of **SI-4** (33 mg, 0.069 mmol, 1 equiv.) in about ½ mL of DCM was added trifluoroacetic acid (TFA, 1.3 mL, 17 mmol, 247 equiv.). The resulting solution was stirred for 1 h and then concentrated to dryness in vacuo. Flash column chromatography on silica using a gradient of 2-30% MeOH in DCM followed by flash column chromatography on silica using a gradient of 2-10% MeCN in water afforded **SI-5** (12 mg, 55%).

^{1}H NMR (500 MHz, methanol-d_4): δ 7.71 (d, *J* = 7.9 Hz, 1H), 7.36 (d, *J* = 8.1 Hz, 1H), 7.21 (s, 1H), 7.12 (ddd, *J* = 8.2, 7.0, 1.2 Hz, 1H), 7.06 (ddd, *J* = 8.0, 7.0, 1.0 Hz, 1H), 4.29 (d, *J* = 5.3 Hz, 1H), 4.20 (dd, *J* = 8.5, 5.3 Hz, 1H), 3.46 (dd, *J* = 15.1, 5.2 Hz, 1H), 3.19 (dd, *J* = 15.1, 8.5 Hz, 1H), 1.95 – 1.85 (m, 1H), 1.62 – 1.51 (m, 1H), 1.22 – 1.11 (m, 1H), 0.97 – 0.88 (m, 6H).

^{13}C NMR (126 MHz, methanol-d_4): δ 170.07, 138.31, 128.42, 125.76, 122.81, 120.24, 119.29, 112.51, 108.31, 61.07, 55.04, 39.07, 28.93, 26.18, 16.32, 12.17.

L-Isoleucinyl-L-tryptophan (SI-7)

To a vial containing *N*-(*tert*-butoxycarbonyl)-L-isoleucine (51 mg, 0.22 mmol, 1.0 equiv.), L-tryptophan *tert*-butyl ester hydrochloride (84 mg, 0.28 mmol, 1.3 equiv.), EDC hydrochloride (142 mg, 0.74 mmol, 3.4 equiv.) and DMAP (54 mg, 0.44 mmol, 2.0 equiv.) was added 4 mL of DCM. The resulting solution was stirred at room temperature overnight and concentrated in vacuo. Flash column chromatography on silica using a gradient of 2-36% ethyl acetate in n-hexane afforded [*N*-(*tert*-butoxycarbonyl)-L-isoleucinyl]-L-tryptophan *tert*-butyl ester (**SI-6**) (101 mg, 97%).

To a solution of **SI-6** (50 mg, 0.11 mmol, 1 equiv.) in about ½ mL of DCM was added TFA (2.0 mL, 26 mmol, 245 equiv.). The resulting solution was stirred for 1 h and then concentrated to dryness in vacuo. Flash column chromatography on silica using a gradient of 2-20% MeCN in water afforded **SI-7** (20 mg, 59%).

^{1}H NMR (500 MHz, methanol-d_4): δ 7.63 (d, *J* = 7.9 Hz, 1H), 7.29 (d, *J* = 8.1 Hz, 1H), 7.13 (s, 1H), 7.05 (t, *J* = 7.5 Hz, 1H), 6.98 (t, *J* = 7.4 Hz, 1H), 4.62 (dd, *J* = 7.7, 5.0 Hz, 1H), 3.55 (d, *J* = 5.3 Hz, 1H), 3.38 (dd, *J* = 14.7, 5.0 Hz, 1H), 3.21 (dd, *J* = 14.7, 7.8 Hz, 1H), 1.88 – 1.79 (m, *J* = 5.7 Hz, 1H), 1.51 – 1.40 (m, 1H), 1.17 – 1.06 (m, 1H), 0.93 (d, *J* = 6.9 Hz, 3H), 0.86 (t, *J* = 7.3 Hz, 3H).

^{13}C NMR (126 MHz, methanol-d_4): δ 177.98, 169.62, 137.96, 129.24, 124.40, 122.16, 119.59, 112.12, 111.98, 59.45, 57.23, 38.18, 29.12, 25.17, 15.26, 11.72.

2. Supplementary Figures

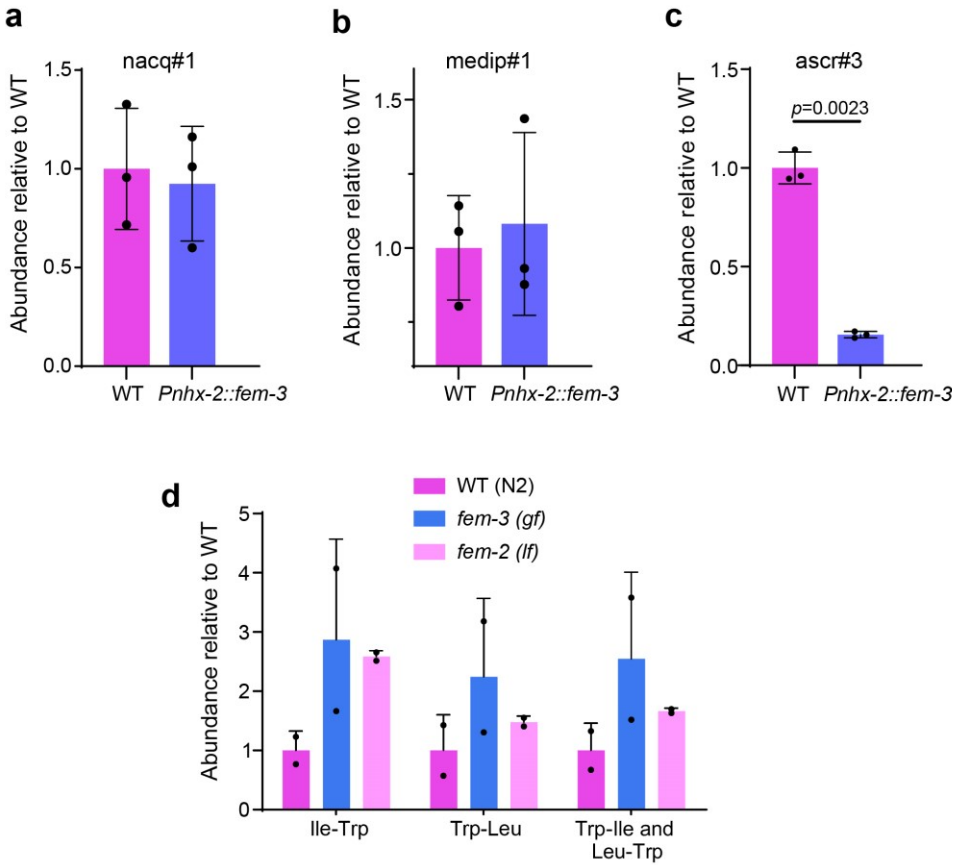

Supplementary Figure 1. Relative abundances of nacq#1, medip#1, and medip#1-related dipeptides in sex mutants. a, b, Relative abundances of nacq#1 (**a**) and medip#1 (**b**) in WT hermaphrodites and intestinally masculinized *Pnhx-2::fem-3* hermaphrodites, *exo*-metabolome. **c**, Relative abundances of ascr#3 in WT hermaphrodites and intestinally masculinized *Pnhx-2::fem-3* hermaphrodites, *exo*-metabolome. **d,** Relative abundances of medip#1-related non-methylated dipeptides in *endo*-metabolome extracts of *fem-3 (gf)* and *fem-2 (lf)* mutants compared to WT hermaphrodites. Bars represent mean ± s.e.m. with two or three independent biological replicates; *p* values were calculated by two-sided Welch *t*-tests. Source data are provided as a Source Data file.

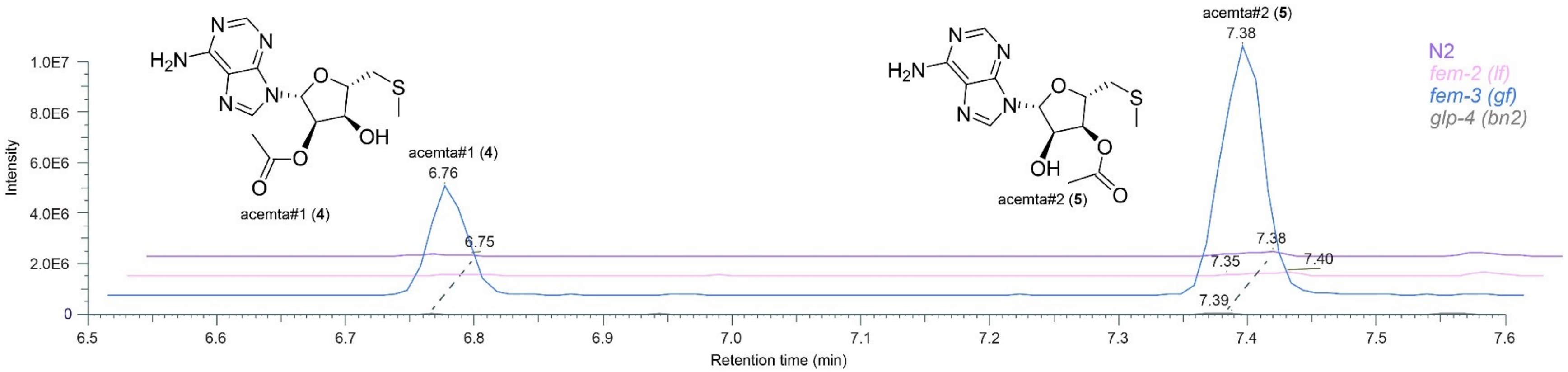

Supplementary Figure 2. Extracted ion chromatograms (EICs) of acemta#1 (4) and acemta#2 (5). EIC (ESI+) of *m/z* 340.1074 in N2 and *him-5* endo-metabolome samples showing peaks for acemta#1 (6.76 min) and acemta#2 (7.38 min) and MS2 spectrum for acemta#2 acquired from a *fem-3 (gf)* endo-metabolome sample.

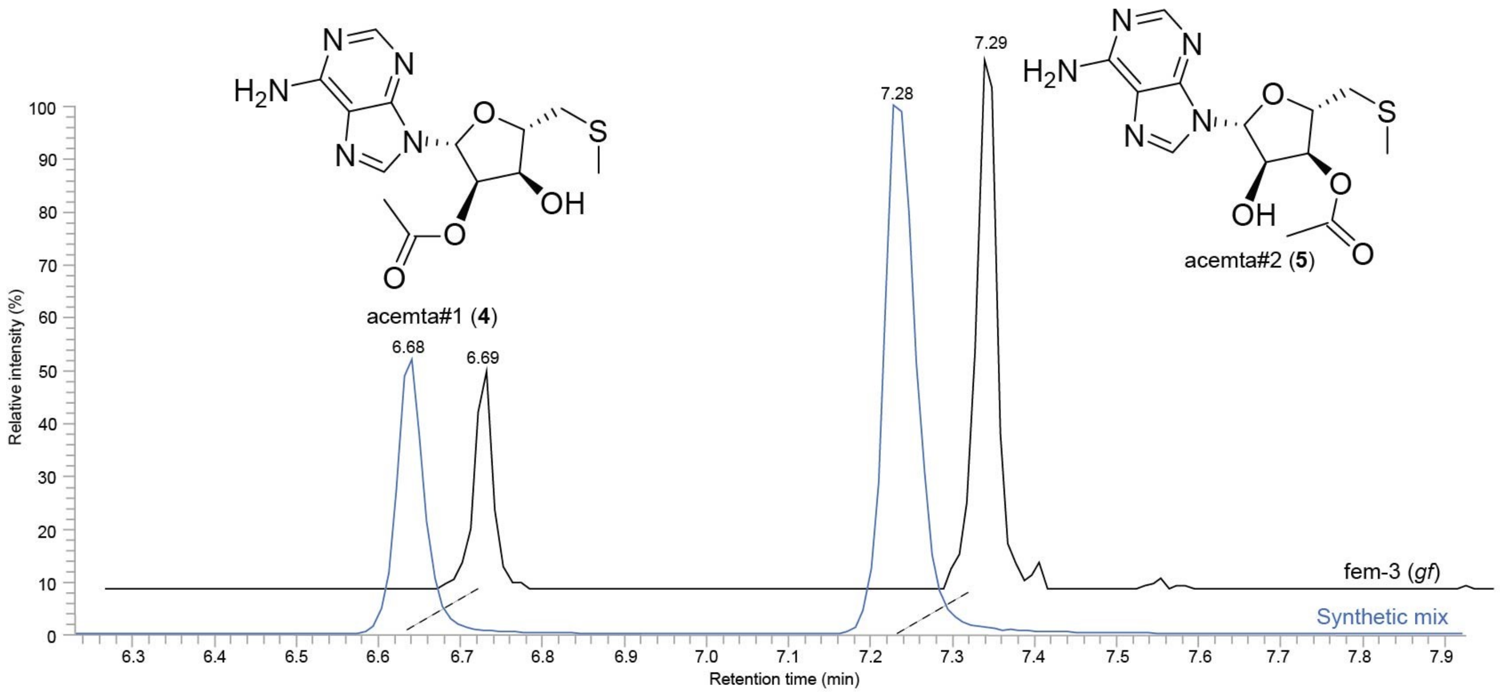

Supplementary Figure 3. EICs of natural and synthetic acemta#1 (4) and acemta#2 (5). EIC (ESI+) of *m/z* 340.1074 in a *fem-3 (gf)* endo-metabolome sample (black) and a synthetic standard containing a mixture of acemta#1 and acemta#2 (blue).

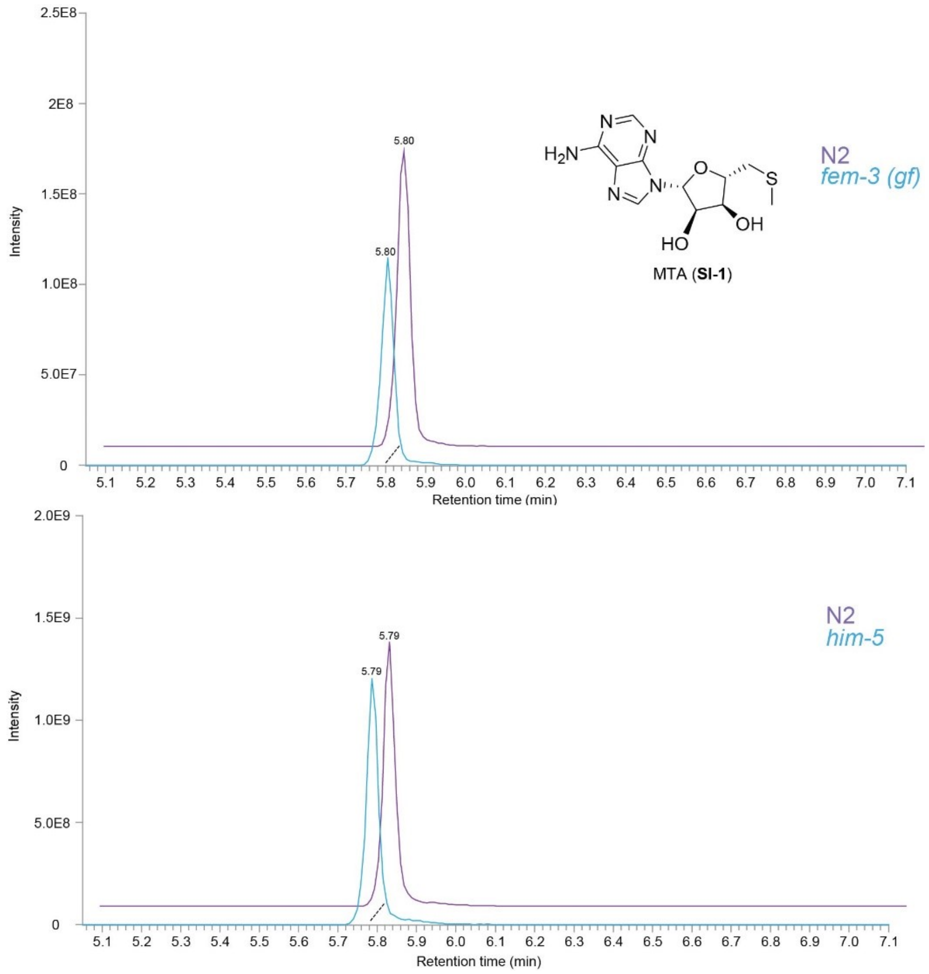

Supplementary Figure 4. The abundance of methylthioadenosine is not sex-dependent. Comparison of signal intensity of putative precursor to acemta derivatives (**4** and **5**), methylthioadenosine (MTA, **SI-1**) for WT, *fem-3 (gf)*, and *him-5* endo-metabolome samples reveals very little differences. Note: the comparisons were done via biological replicates of WT *C. elegans*, notable by the differences in signal intensity between samples.

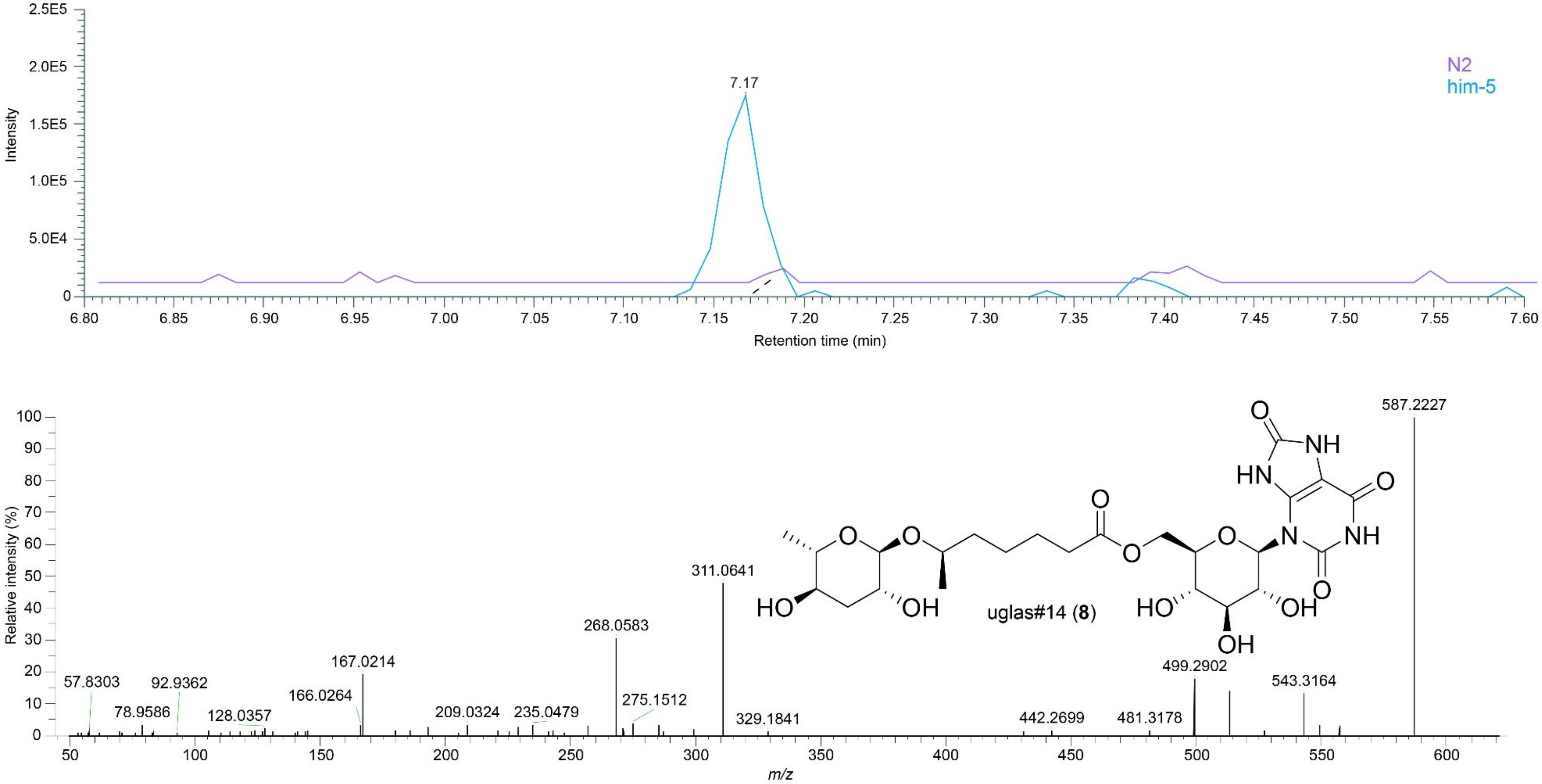

Supplementary Figure 5. EICs and MS2 spectrum of uglas#14 (8). EIC (ESI-) of *m/z* 587.2206 in WT and *him-5* endo-metabolome samples showing peaks for uglas#14 and MS2 spectrum (ESI-) for uglas#14 acquired from a *him-5* endo-metabolome sample.

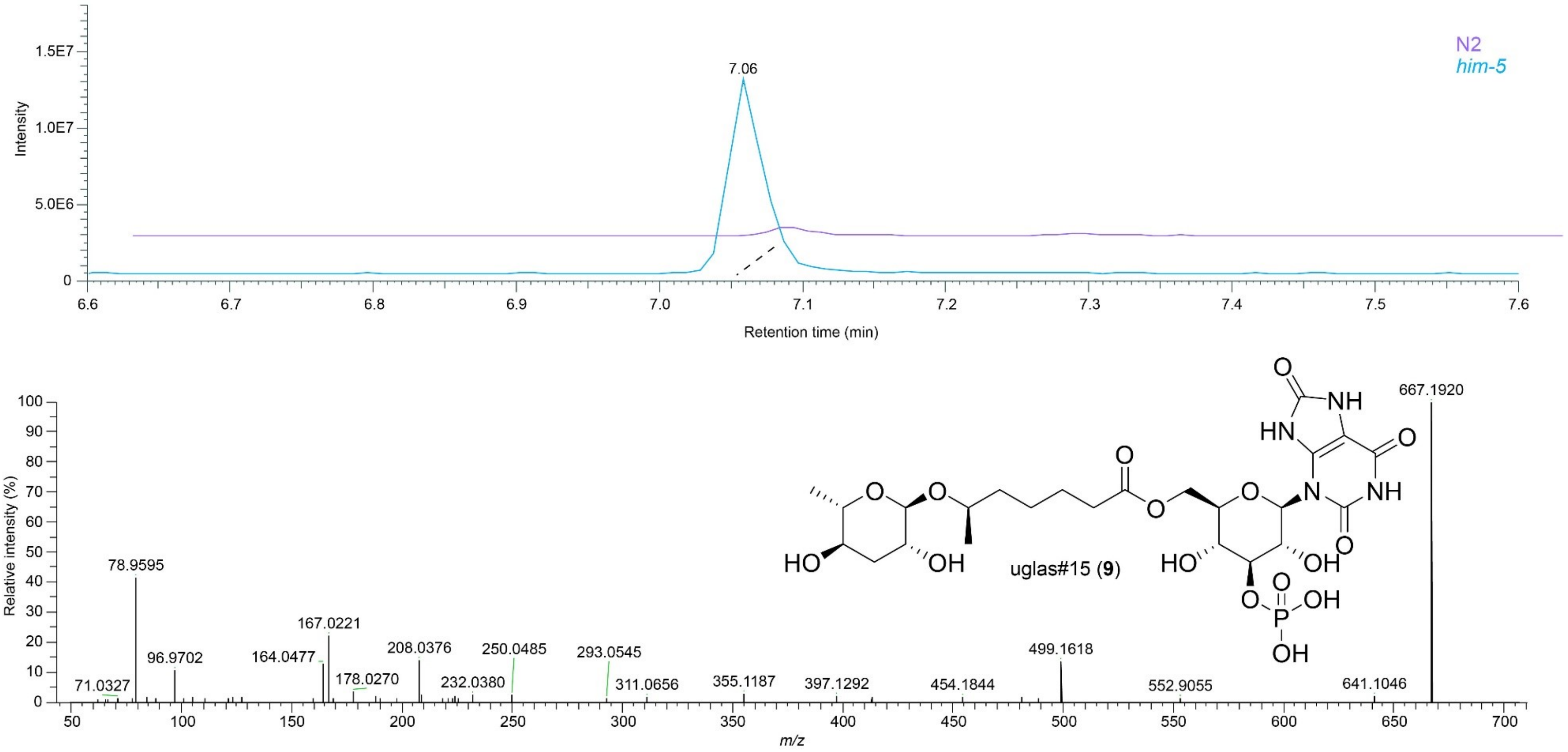

Supplementary Figure 6. EICs and MS2 spectrum of uglas#15 (9). EIC (ESI-) of *m/z* 667.1869 in WT and *him-5* endo-metabolome samples showing peaks for uglas#15 and MS2 spectrum (ESI-) for uglas#15 acquired from a *him-5* endo-metabolome sample.

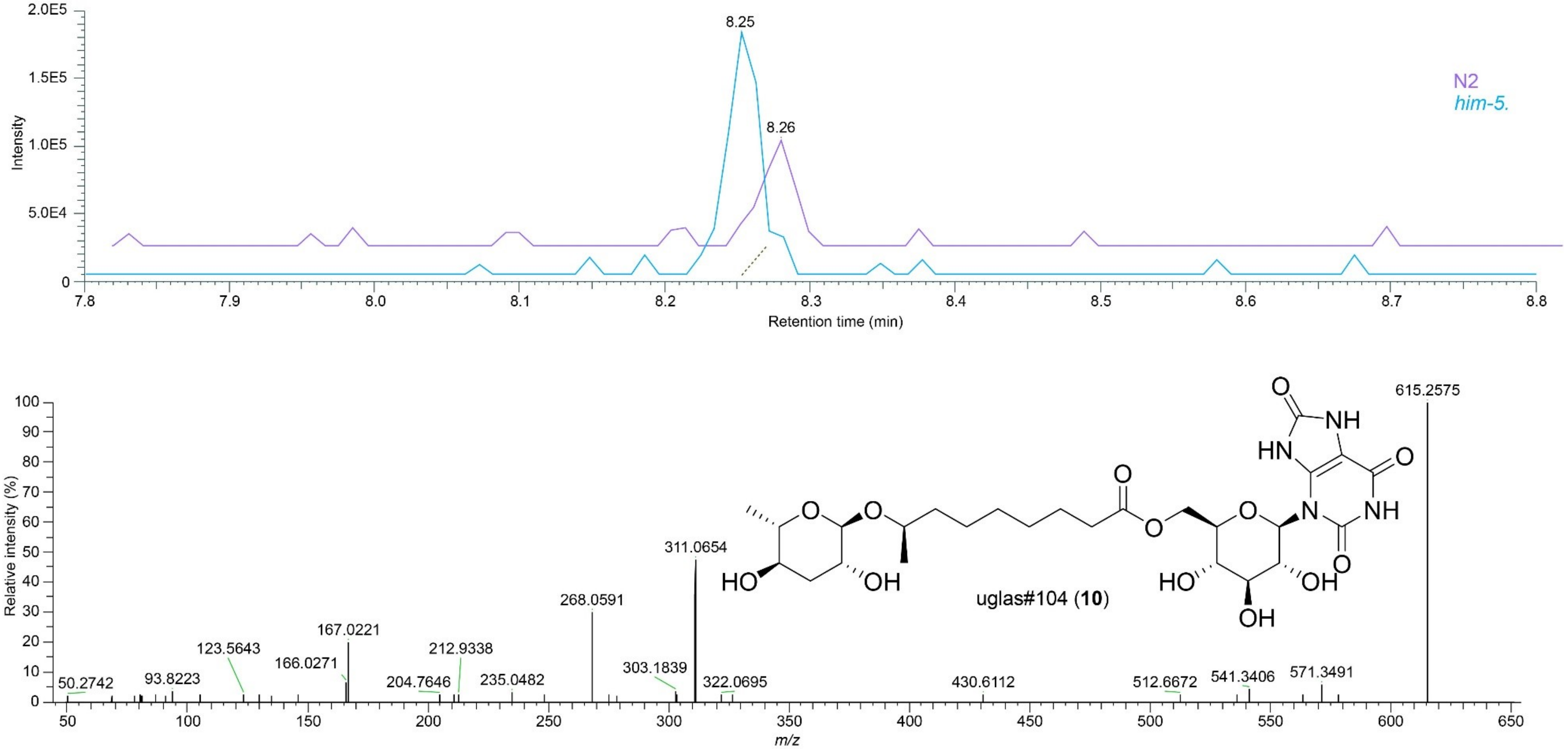

Supplementary Figure 7. EICs and MS2 spectrum of uglas#104 (10). EIC (ESI-) of *m/z* 615.2519 in WT and *him-5* endo-metabolome samples showing peaks for uglas#104 and MS2 spectrum (ESI-) for uglas#104 acquired from a *him-5* endo-metabolome sample.

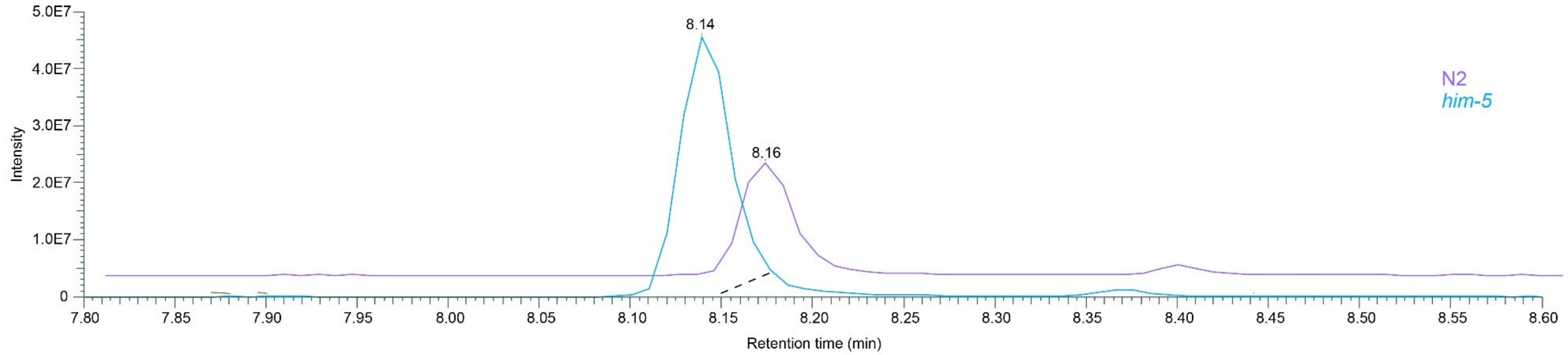

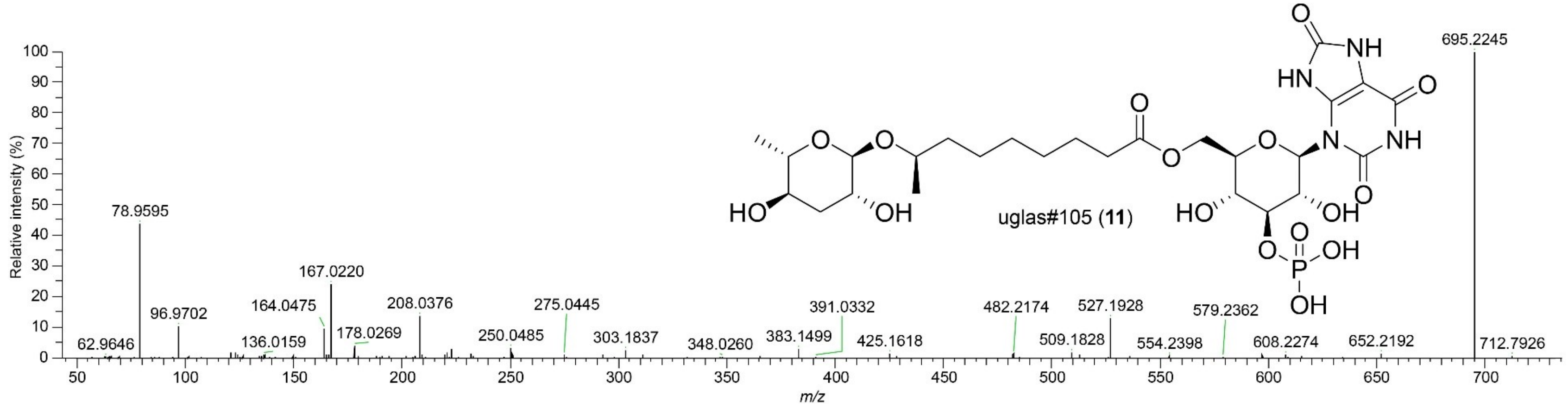

Supplementary Figure 8. EICs and MS2 spectrum of uglas#105 (11). EIC (ESI-) of *m/z* 695.2182 in WT and *him-5* endo-metabolome samples showing peaks for uglas#105 and MS2 spectrum (ESI-) for uglas#105 acquired from a *him-5* endo-metabolome sample.

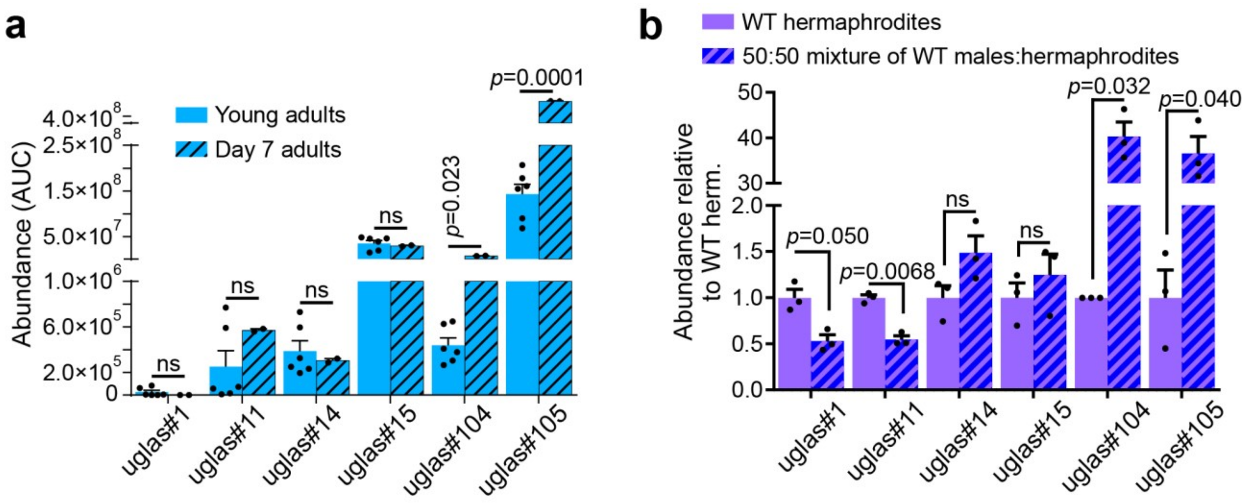

Supplementary Figure 9. Relative abundances of uglas-family metabolites in the *endo*-metabolomes. **a**, Young adults compared to day-7 adults. **b**, Small plate-based samples of WT hermaphrodites and 50:50 mixtures of males and hermaphrodites. Bars represent mean ± s.e.m. with six (**a**, young adults), two (**a**, day-7 adults), or three (**b**) independent biological replicates; *p* values were calculated by two-sided Welch *t*-tests with Holm-Šídák correction; ns, not significant. Source data are provided as a Source Data file.

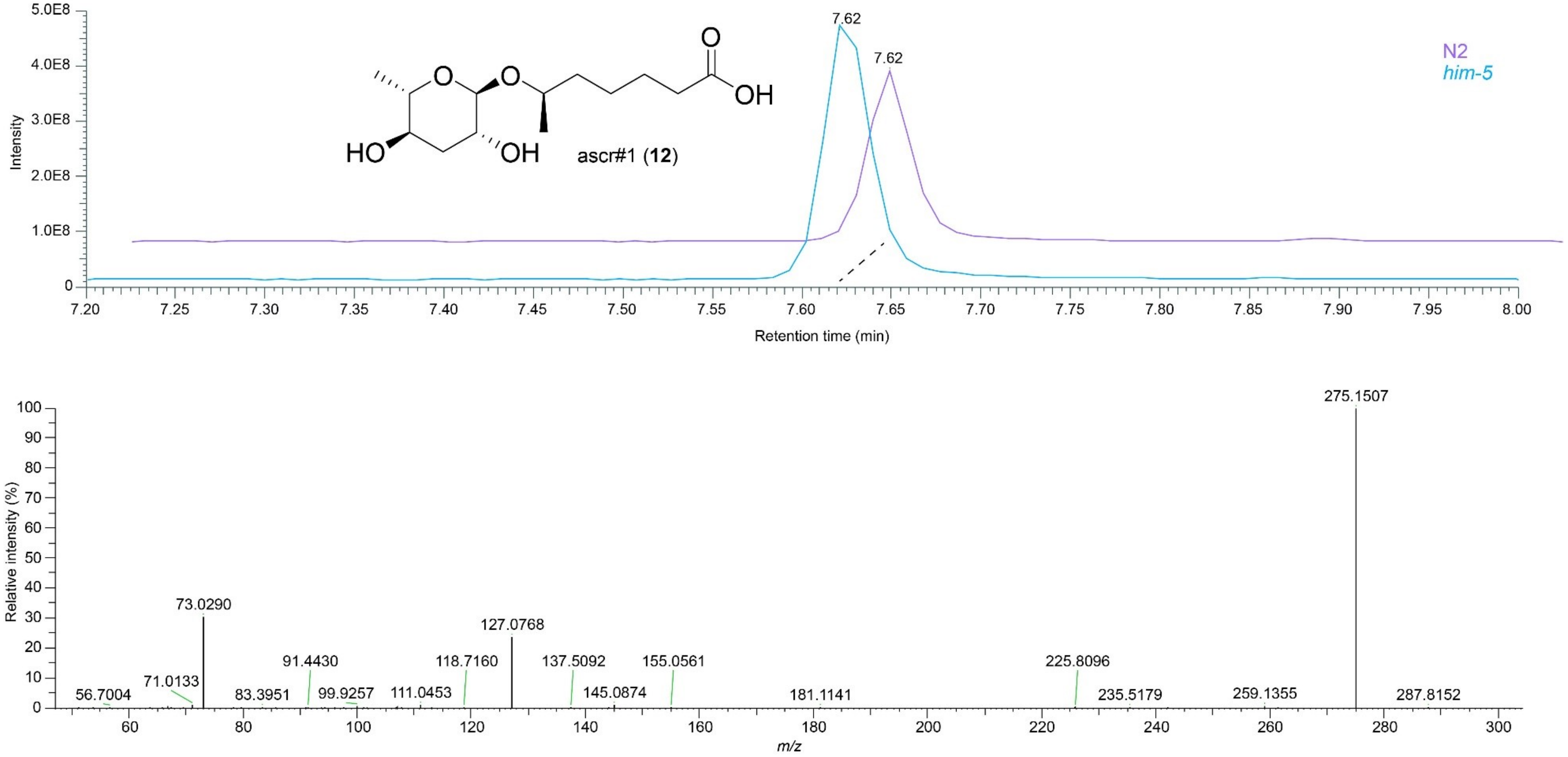

Supplementary Figure 10. EICs and MS2 spectrum of ascr#1 (12). EIC (ESI-) of *m/z* 275.1500 in WT and *him-5* exo-metabolome samples showing peaks for ascr#1 and MS2 spectrum (ESI-) for ascr#1 acquired from a *him-5* exo-metabolome sample.

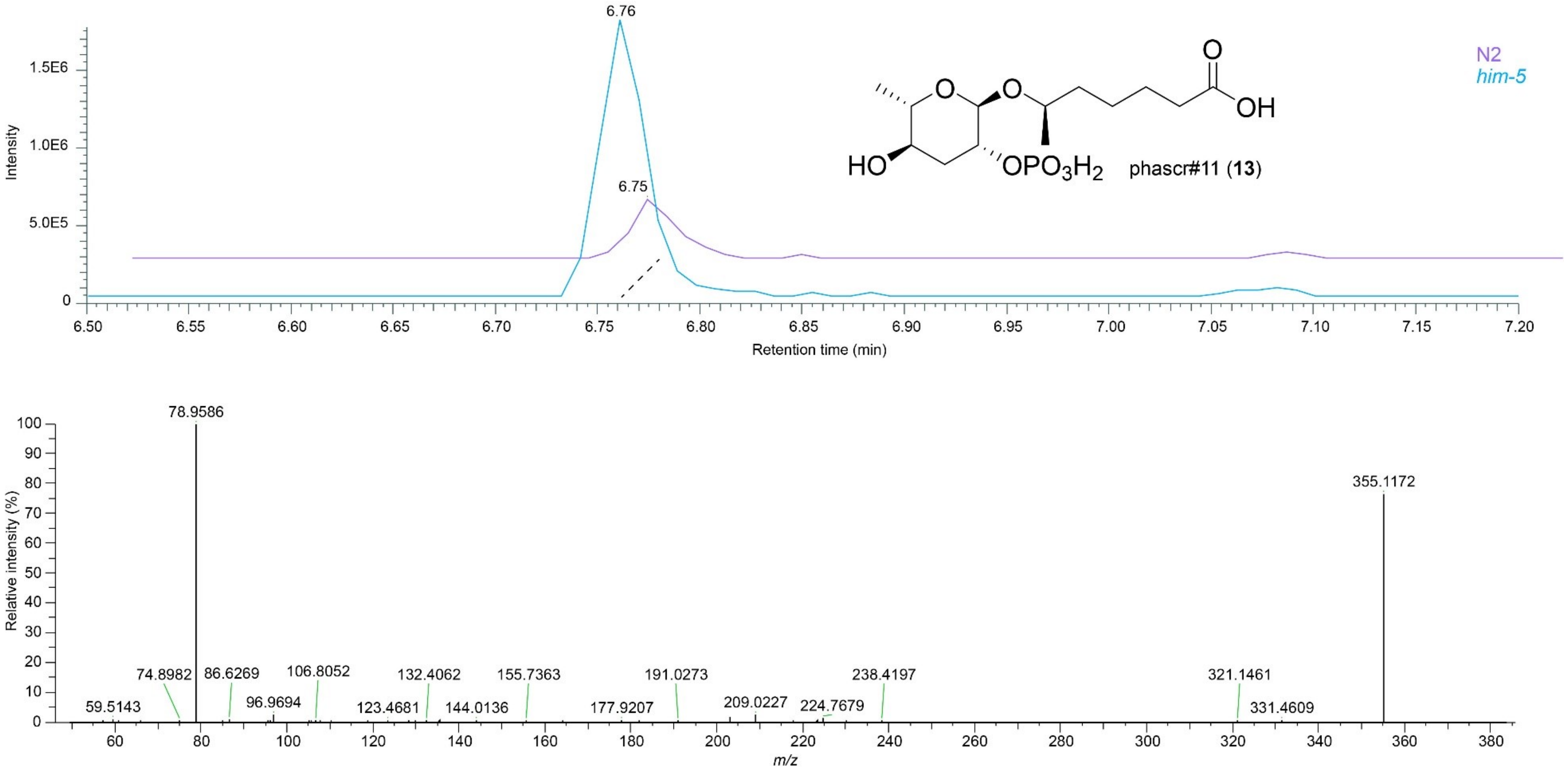

Supplementary Figure 11. EICs and MS2 spectrum of phascr#11 (13). EIC (ESI-) of *m/z* 355.1168 in WT and *him-5* exo-metabolome samples showing peaks for phascr#11 and MS2 spectrum (ESI-) for phascr#11 acquired from a *him-5* exo-metabolome sample[10].

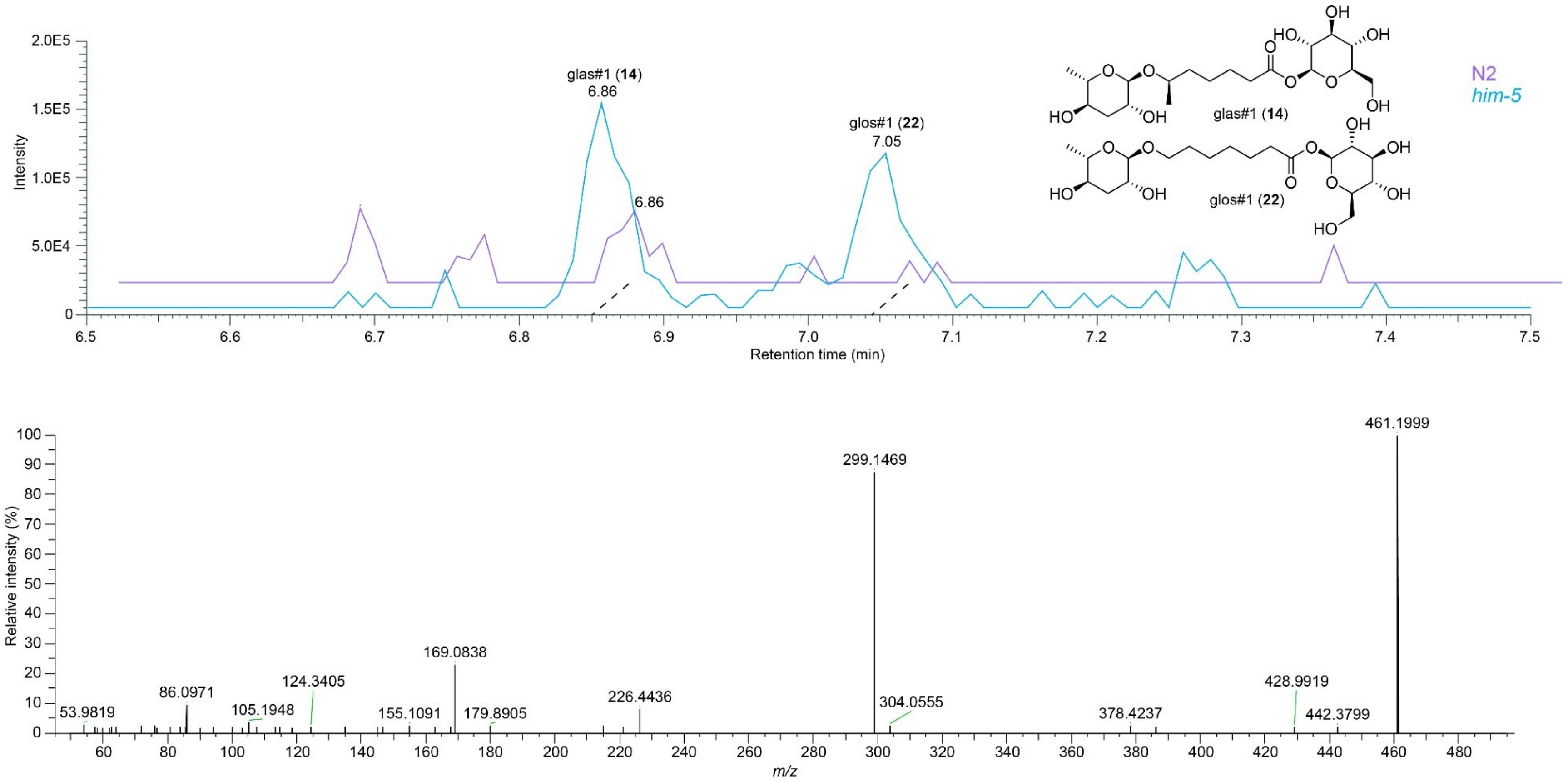

Supplementary Figure 12. EICs and MS2 spectrum of glas#1 (14) and glos#1 (22). EIC (ESI+) of *m/z* 461.1995 in WT and *him-5* endo-metabolome samples showing peaks for glas#1 and glos#1, and MS2 spectrum (ESI+) for glas#1 acquired from a *him-5* endo-metabolome sample.

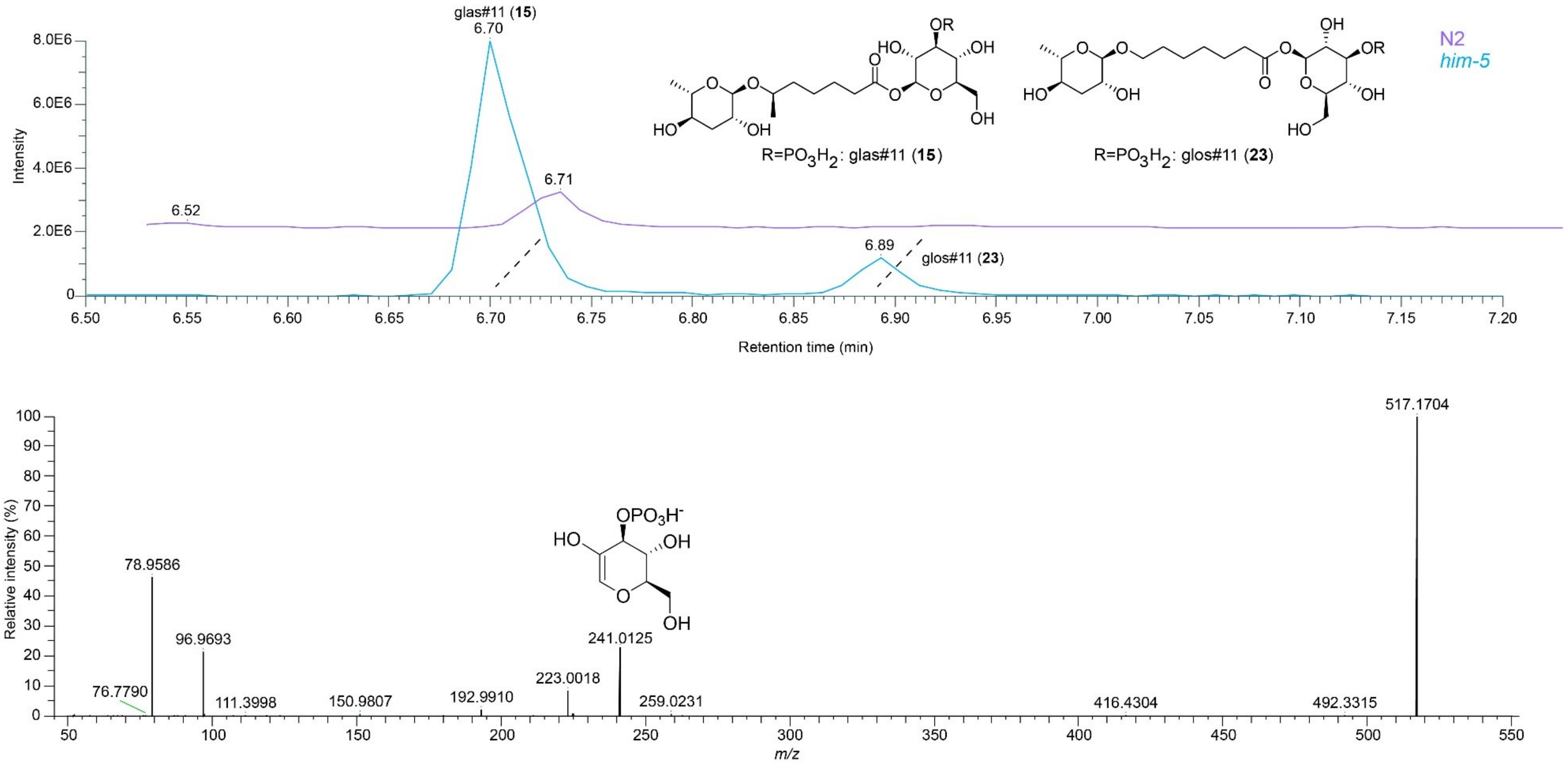

Supplementary Figure 13. EICs and MS2 spectrum of glas#11 (15) and glos#11 (23). EIC (ESI-) of *m/z* 517.1669 in WT and *him-5* exo-metabolome samples showing peaks for glas#11 and glos#11 and MS2 spectrum (ESI-) for glas#11 acquired from a *him-5* exo-metabolome sample. A key fragment suggesting phosphorylation on the glucose moiety is shown for *m/z* = 241.0125.

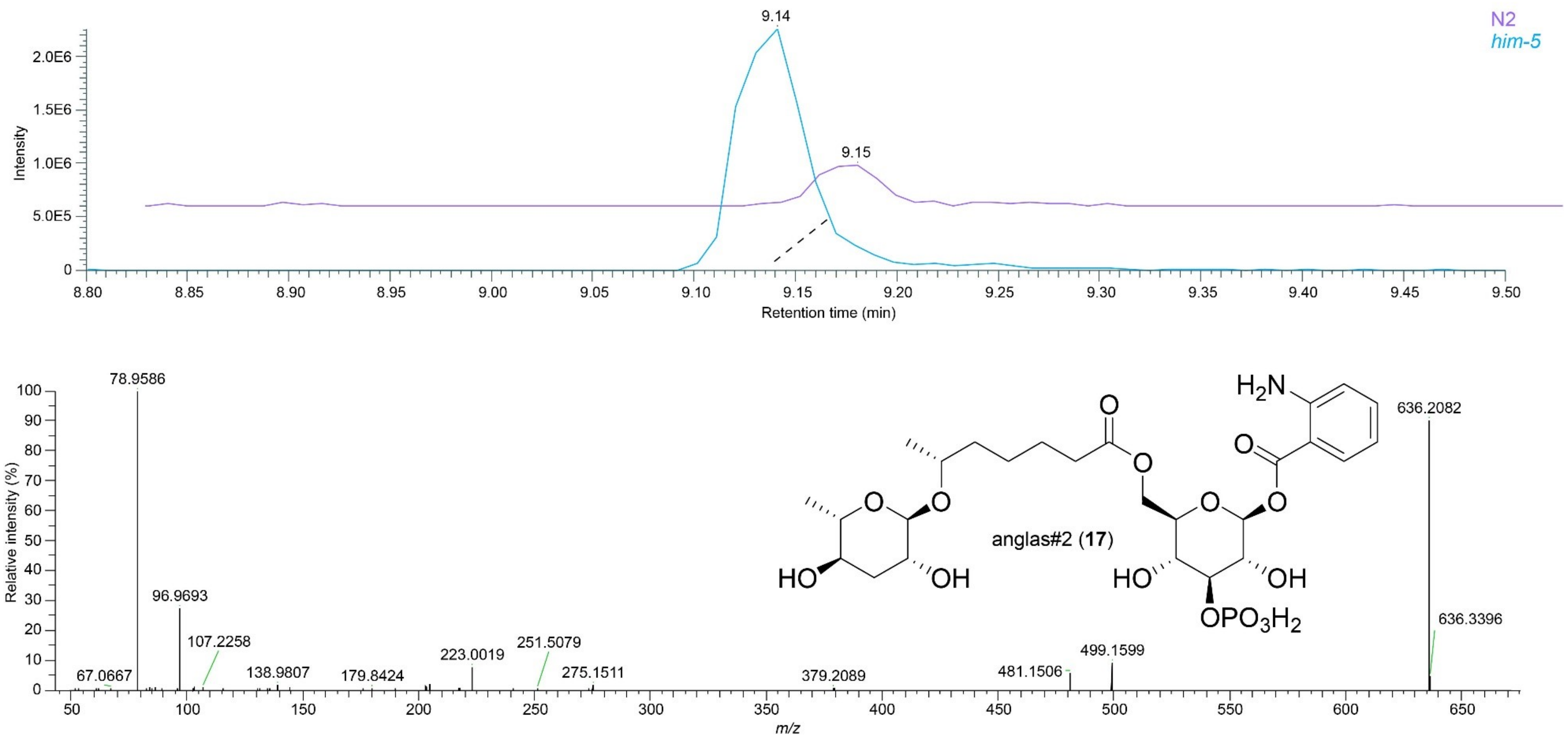

Supplementary Figure 14. EICs and MS2 spectrum of anglas#2 (17). EIC (ESI-) of *m/z* 636.2070 in WT and *him-5* endo-metabolome samples showing peaks for anglas#2 and MS2 spectrum (ESI-) for anglas#2 acquired from a *him-5* endo-metabolome sample.

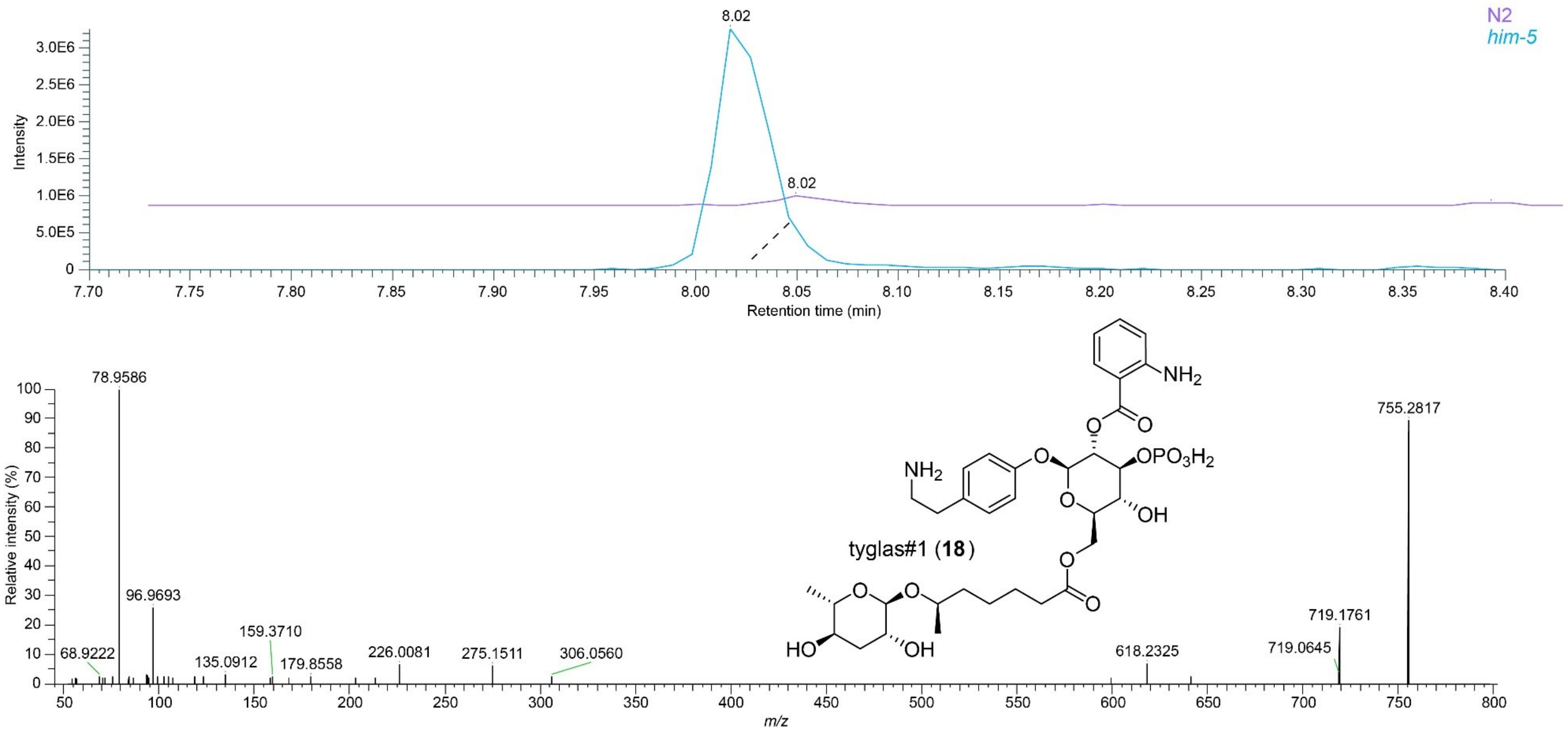

Supplementary Figure 15. EICs and MS2 spectrum of tyglas#1 (18). EIC (ESI-) of *m/z* 755.2807 in WT and *him-5* endo-metabolome samples showing peaks for tyglas#1 and MS2 spectrum (ESI-) for tyglas#1 acquired from a *him-5* endo-metabolome sample See reference for ESI+ MS2 spectrum[11].

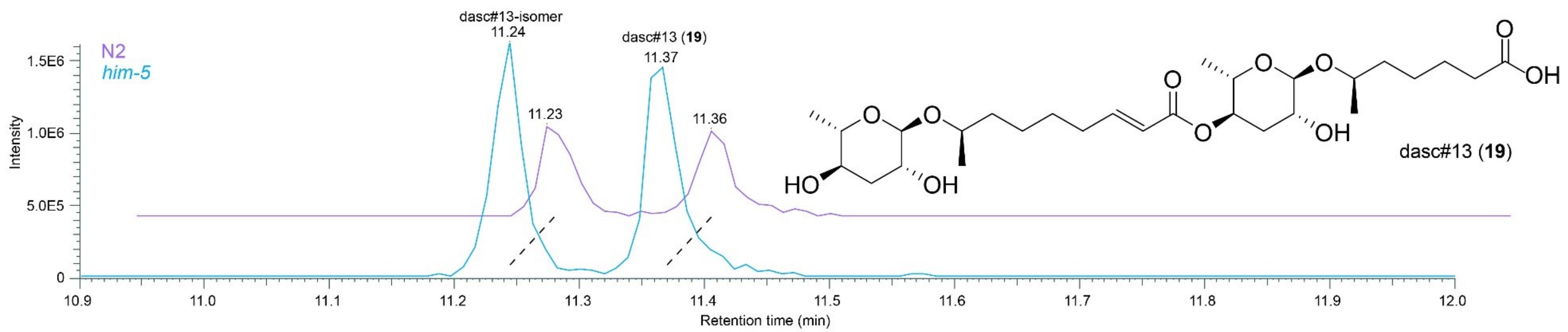

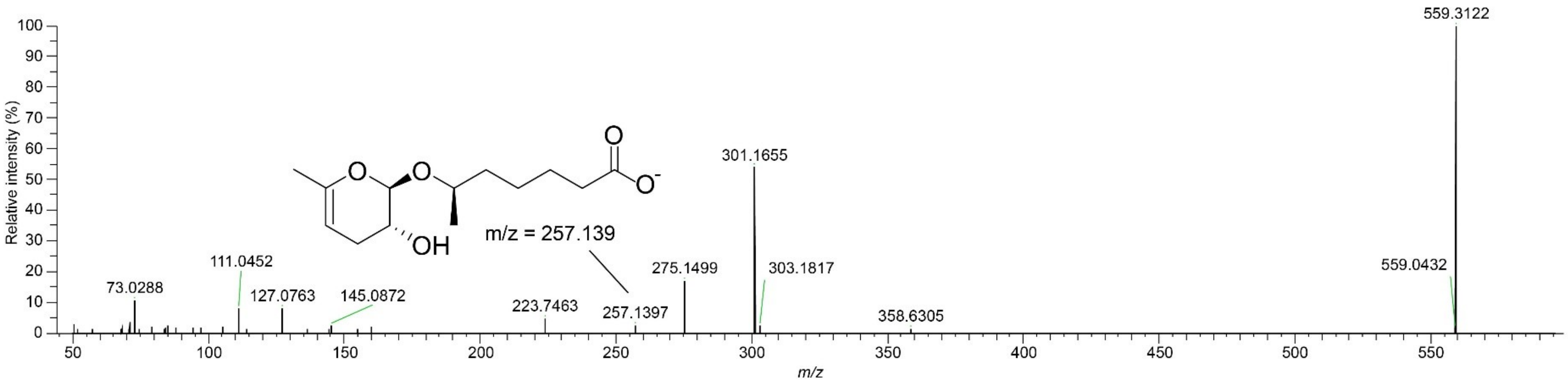

Supplementary Figure 16. EICs and MS2 spectrum of dasc#13 (19). EIC (ESI-) of *m/z* 559.3129 in WT and *him-5* exo-metabolome samples showing peaks for dasc#13 (11.37 min) and MS2 spectrum (ESI-) for dasc#13 (**19**) acquired from a *him-5* exo-metabolome sample. The earlier eluting isomer (11.2 min) is a dasc#13 structural isomer via MS2 analysis. Key fragment (*m/z* = 257.1397) is displayed, suggesting the loss of ascr#3 via elimination at either the 2' or 4'-position (displayed as 4').

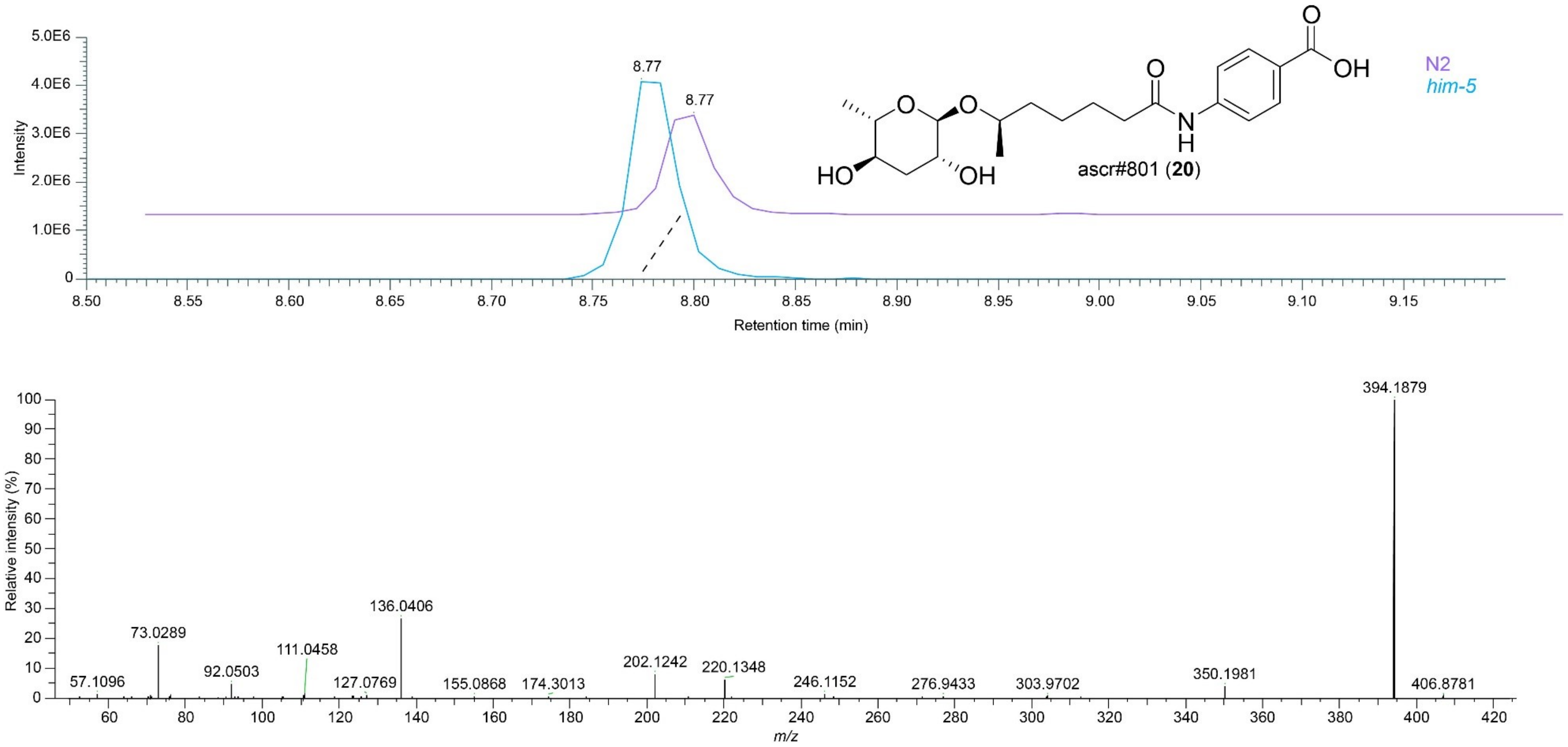

Supplementary Figure 17. EICs and MS2 spectrum of ascr#801 (20). EIC (ESI-) of *m/z* 394.1875 in WT and *him-5* exo-metabolome samples showing peaks for ascr#801 and MS2 spectrum (ESI-) for ascr#801 acquired from a *him-5* exo-metabolome sample.

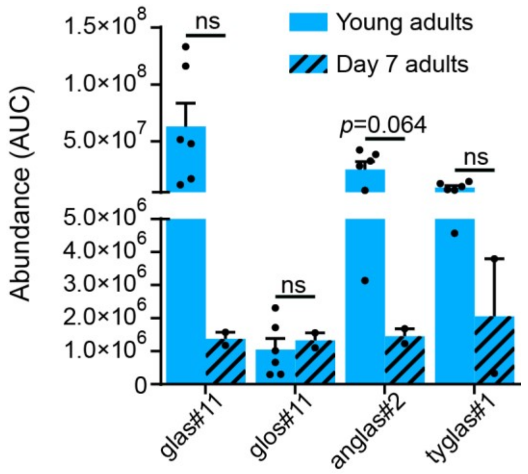

Supplementary Figure 18. Male-enriched metabolites in older hermaphrodites. Abundances of *him-5*-enriched ascr#1 derivatives trended lower in *endo*-metabolome samples from day-7 hermaphrodite adults compared to young hermaphrodite adults, approaching significance in the case of anglas#2. Bars represent mean ± s.e.m. with six (young adults) or two (day 7 adults) independent biological replicates; *p* values were calculated by two-sided Welch *t*-tests with Holm-Šídák correction; ns, not significant. Source data are provided as a Source Data file.

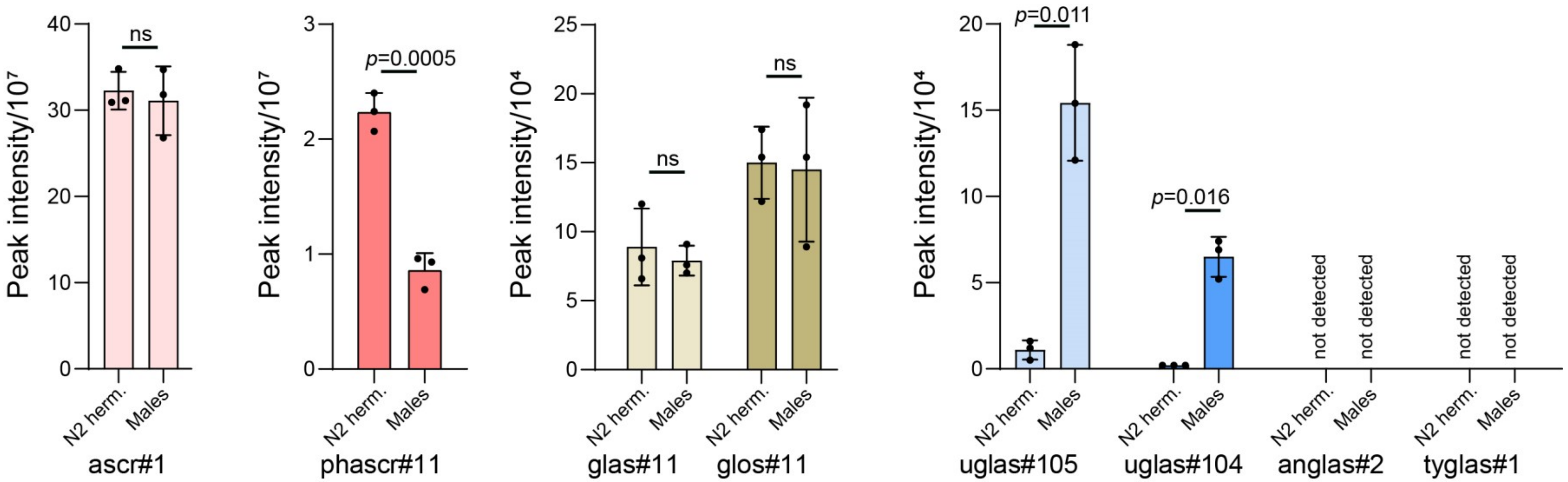

Supplementary Figure 19. Male-enriched metabolites in hand-picked *C. elegans* samples. ascr#1 derivatives are not enriched (ascr#1, phascr#11, glas#11, glos#11) or not detected (anglas#2, tyglas#1) in *endo*-metabolome samples of hand-picked, pure males compared to pure hermaphrodites. In contrast, uglas#104 and uglas#105 are enriched in *endo*-metabolome samples of hand-picked, pure males compared to pure hermaphrodites. Bars represent mean ± s.e.m. with three independent biological replicates; *p* values were calculated by two-sided Welch *t*-tests; ns, not significant. Source data are provided as a Source Data file.

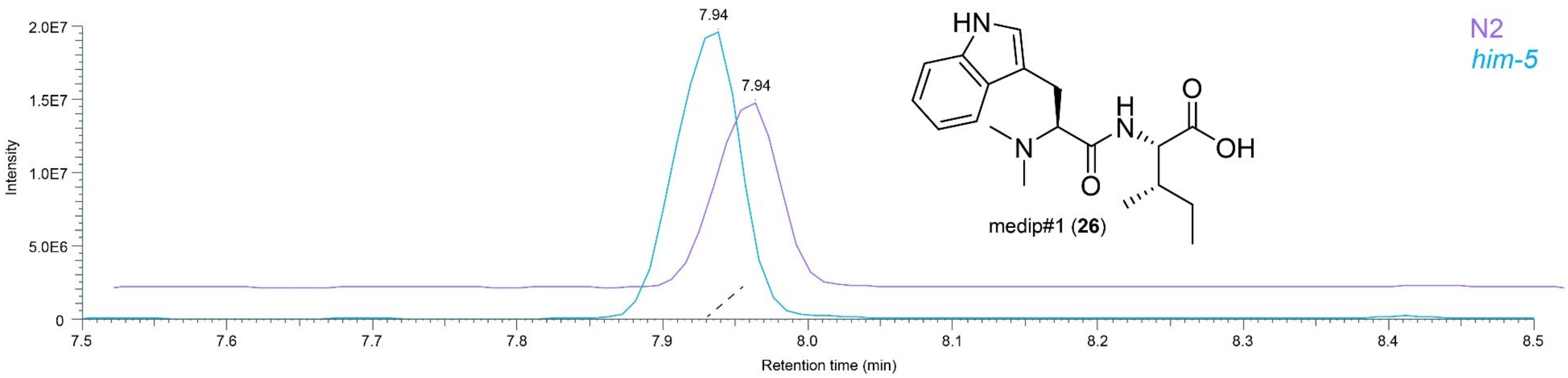

Supplementary Figure 20. Detection of medip#1 in exo-metabolome samples. EICs (ESI+) of *m/z* 346.2125 in WT and *him-5* exo-metabolome samples showing peaks for medip#1.

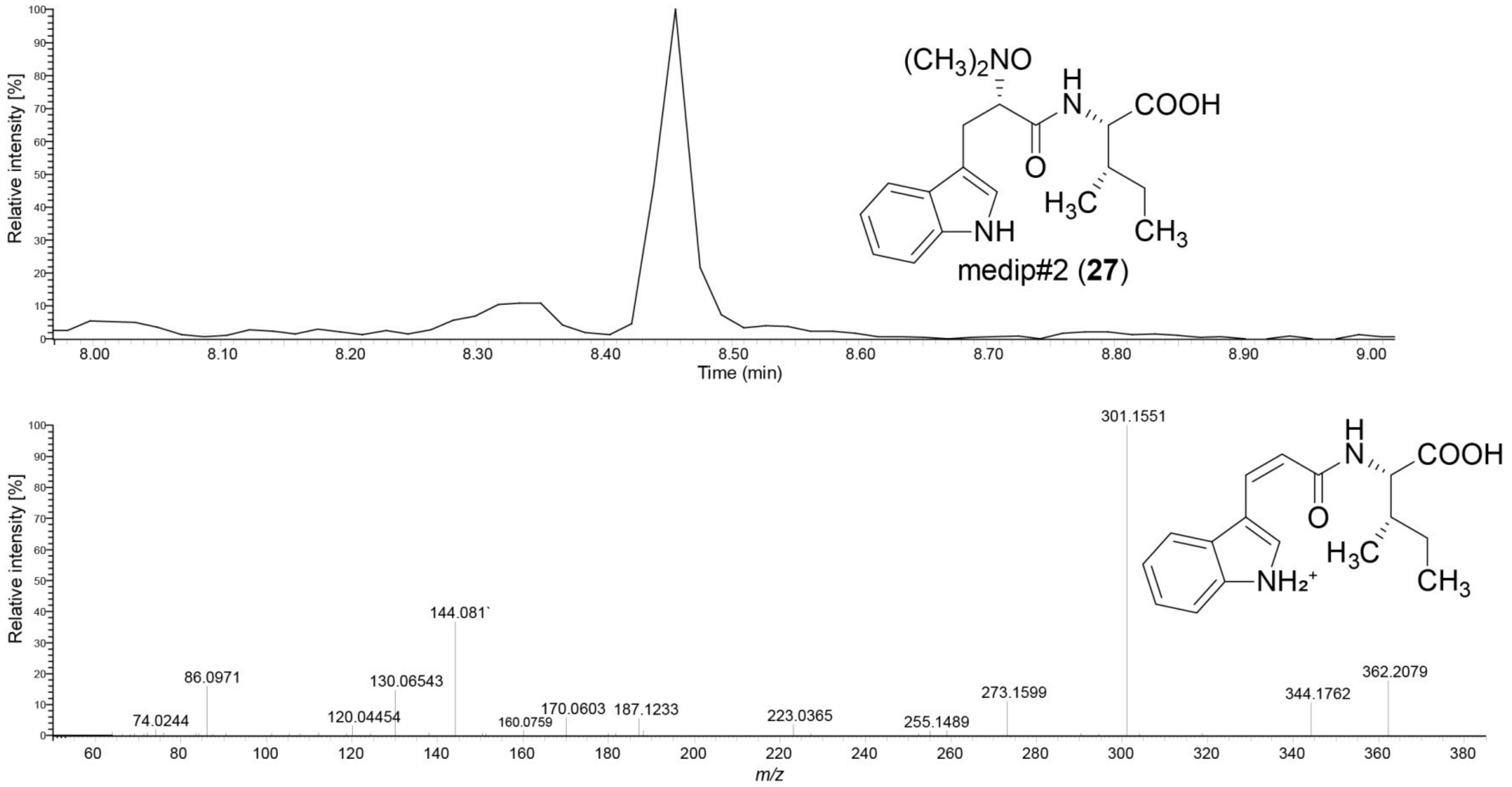

Supplementary Figure 21. EIC and MS2 spectrum of medip#2 (27). EIC (ESI+) of *m/z* 362.2074 in WT and *him-5* exo-metabolome samples showing a peak for medip#2 (top), and MS2 spectrum (ESI+) for medip#2 acquired from a *him-5* exo-metabolome sample showing key fragment at *m/z* 301.1551.

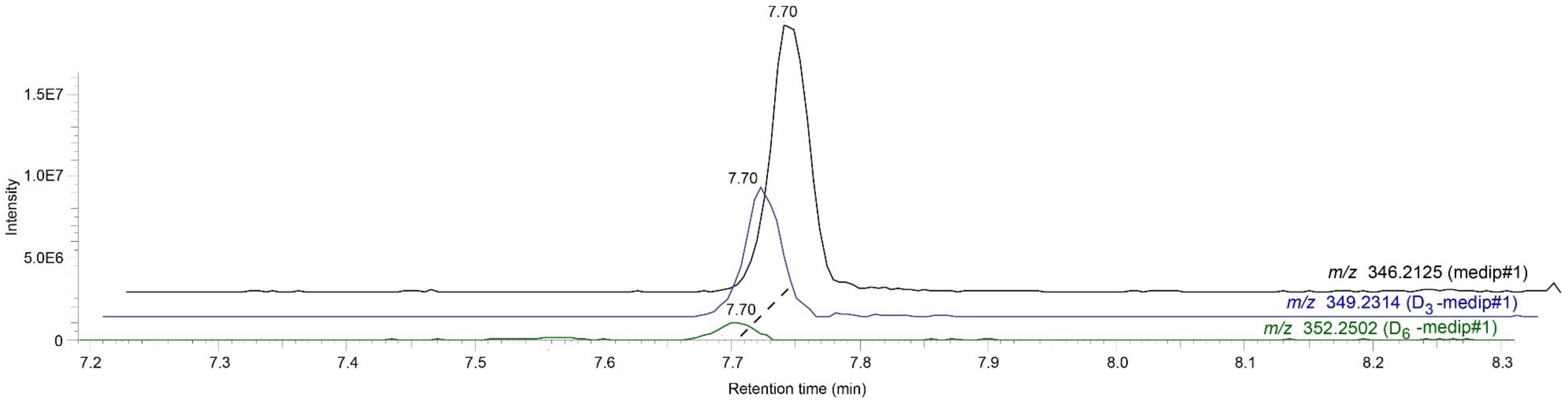

Supplementary Figure 22. D_3-Methionine labeling of medip#1. EICs (ESI+) for medip#1 (black), D_3-medip#1 (blue), D_6-medip#1 (green) in the exo-metabolome of *him-5* animals fed D_3-methionine.

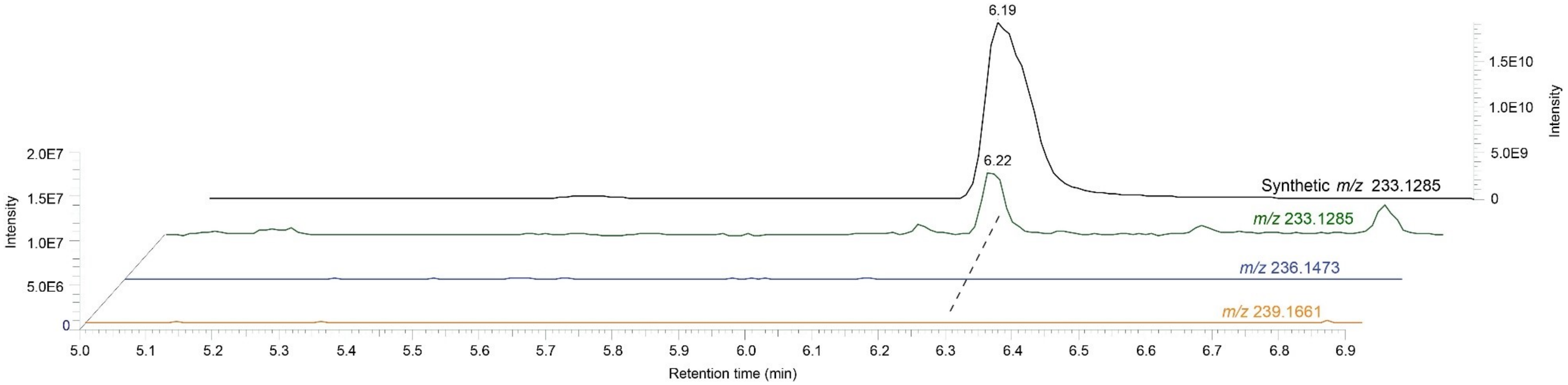

Supplementary Figure 23. D_3-Methionine feeding does not label dimethyltryptophan. EICs (ESI+) of synthetic dimethyltryptophan (black), unlabeled dimethyltryptophan in the *him-5* exo-metabolome (green), the trace for the *m/z* of D_3-dimethyltryptophan, lacking a co-eluting peak (blue), and the trace for the *m/z* of D_6- dimethyltryptophan (orange), also lacking a co-eluting peak.

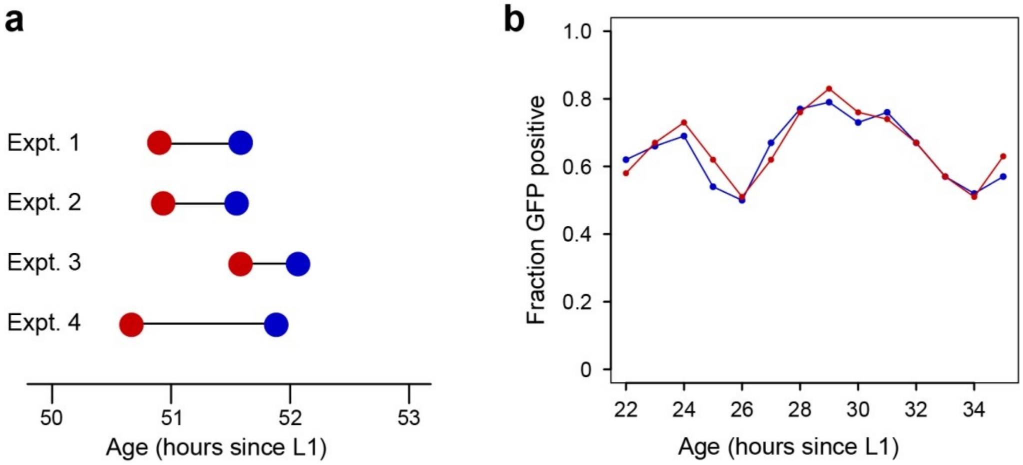

Supplementary Figure 24. Developmental assays with medip#1. a. Time to sexual maturation of hermaphrodites raised on medip#1-conditioned plates (red) *vs.* paired control (blue). Median time of morphologically-defined adulthood is shown for four independent experiments. In Experiments 1, 3, and 4, medip#1 was at 2 nM, while in Experiment 2, it was at 200 nM. In all experiments, n = 23-25 for each condition. **b.** Larval development during L1-L3 stages does not show acceleration on medip#1. Fractions of GFP-positive *mlt-10::GFP* hermaphrodites raised on 2 nM medip#1 (red) or control (blue). At none of the points was there a significant difference between the two curves (Binomial test with the Bonferroni correction). n = 342 for control animals and 336 medip#1 animals. Six biologically independent plates each for control and medip#1 were monitored every hour. Each reported data point is the average fraction of GFP positive animals across the six plates. In all experiments in **a** and **b**, it was not known to the investigator which plates were medip#1-conditioned and which were control. Source data are provided as a Source Data file.

.

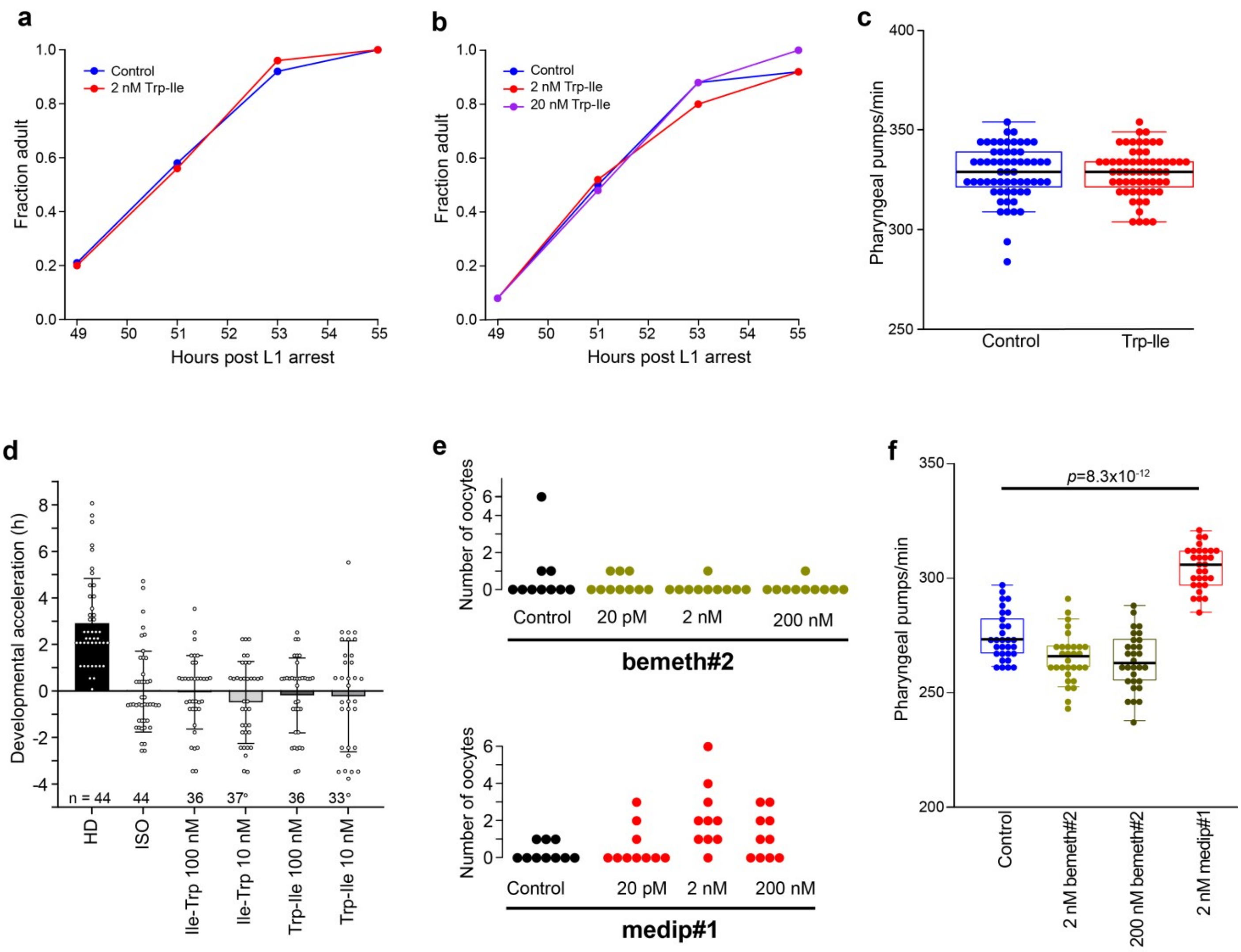

Supplementary Figure 25. medip#1-related dipeptides and bemeth#2 do not accelerate development or increase pharyngeal pumping. a. No acceleration of larval development was observed on 2 nM of the Trp-Ile dipeptide (red) compared to control (blue). Data are from one experiment with n = 24 animals for control, n = 25 for 2 nM Trp-Ile. **b.** No acceleration of larval development was observed on 2 nM (red) or 20 nM (purple) of the Trp-Ile dipeptide compared to control (blue). Data are from one experimental replicate with n = 24 for control, n = 25 for each of the two concentrations of Trp-Ile. The 2 nM experiment in this panel is an independent replicate of the experiment shown in panel a. **c.** The frequency of pharyngeal pumping was not increased on 2 nM of the Trp-Ile dipeptide (red) compared to control (blue). Data are from one experiment with n = 60 animals for each condition. Boxes represent the two inner quartiles, horizontal lines represent medians, whiskers extend to 1.5X of the box data. **d.** Time point of first egg laying of isolated worms on different concentrations of Trp-Ile and Ile-Trp compared to untreated isolated worms (ISO, control) and grouped worms (high density, HD). Bars represent mean ± s.e.m. Data are from four independent biological replicates, with the total number of animals indicated above the x-axis. **e.** No acceleration of larval development was observed at any of the three shown concentrations of bemeth#2

(green) compared to control (black). In contrast, at two higher concentrations (2 nM and 200 nM) of medip#1 (red) we did observe acceleration compared to control (black), as described in the main text. For each condition, data are from one experiment with n = 10 animals. **f.** The frequency of pharyngeal pumping was not increased on either of the two tested concentrations of bemeth#2 (green) compared to control (blue). For comparison, on 2 nM of medip#1 (red) there was significant increase in the frequency of pharyngeal pumping (medip#1 data are from Figure 4k). For each condition, data are from one experiment with n = 30 animals. Boxes represent the two inner quartiles, horizontal lines represent medians, whiskers extend to 1.5X of the box data. In **c**, **e**, **f**, each circle represents a single tested animal; *p* value was calculated by Kolmogorov-Smirnov test. Source data are provided as a Source Data file.

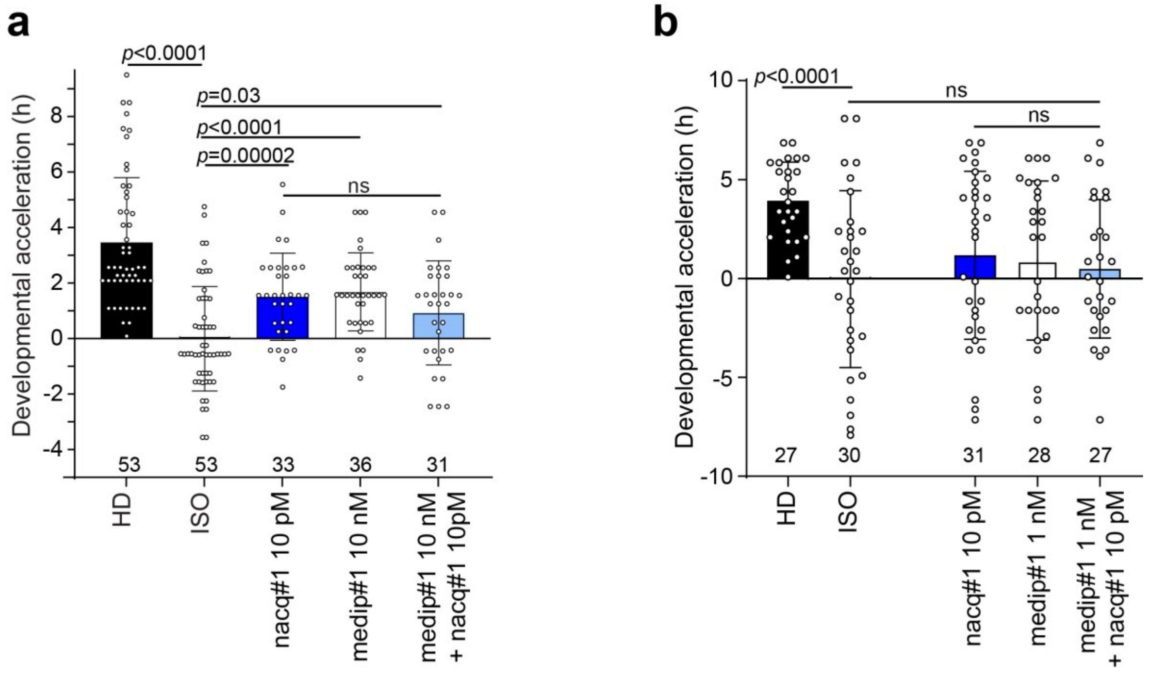

Supplementary Figure 26. Developmental acceleration of a mixture of medip#1 and nacq#1. a, b, Time point of first egg laying of isolated worms on the indicated concentrations of medip#1, nacq#1, or mixtures of medip#1 and nacq#1, compared to untreated isolated worms (ISO, control) and grouped worms (high density, HD). Bars represent mean ± s.e.m. Data are from four independent biological replicates, with the total number of animals used for each condition indicated above the x-axis; *p* values were calculated by two-sided Welch *t*-tests; ns, not significant. Source data are provided as a Source Data file.

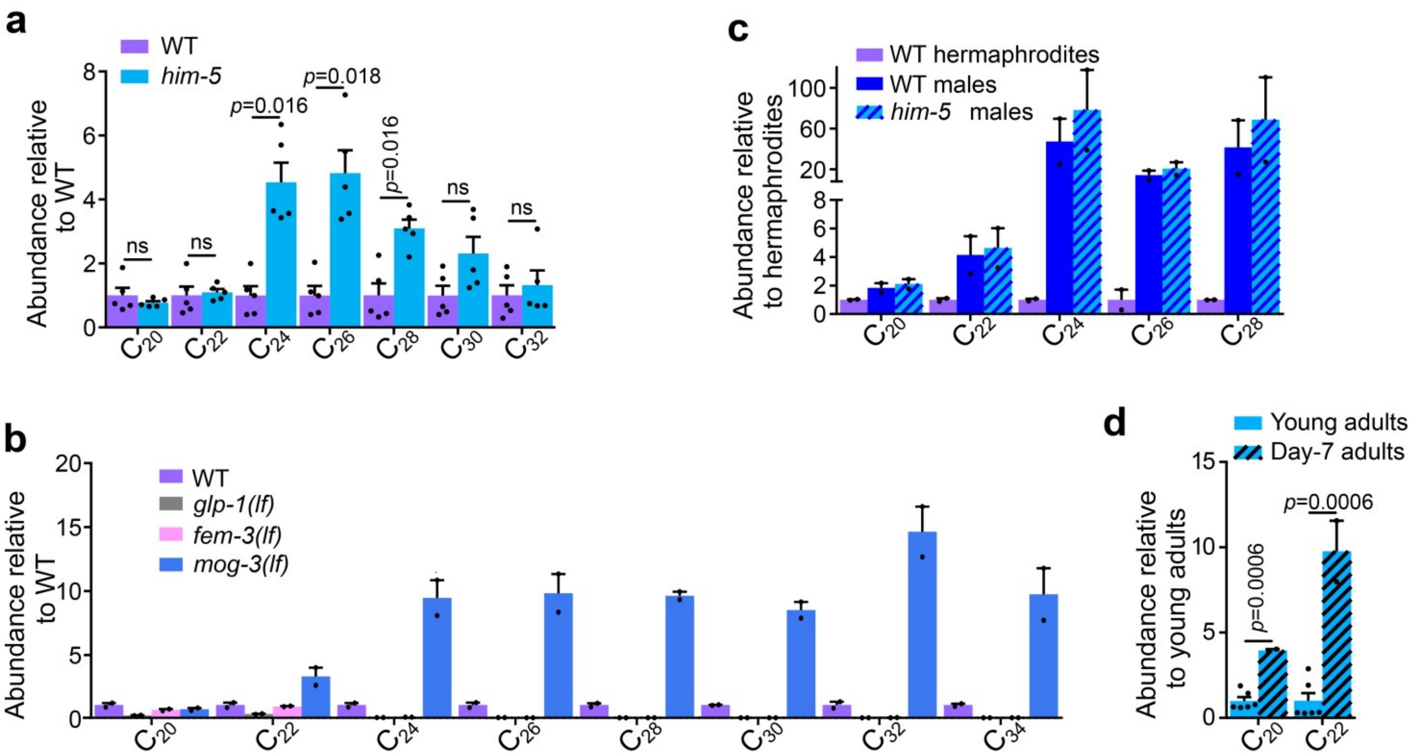

Supplementary Figure 27. Male germline-dependent increase in polyunsaturated very long chain fatty acids (VLCPUFAs). a. VLCPUFAs with 20-32 carbons were significantly enriched in *him-5* cultures. Bars represent mean ± s.e.m. with five independent biological replicates; *p* values were calculated by two-sided Welch *t*-tests with Holm-Šídák correction; ns, not significant. **b.** VLCPUFAs with 24-32 carbons were enriched in germline-masculinized *mog-3(lf)* animals compared to WT, but absent in *glp-1 (lf)*and *fem-3(lf)* animals. Note that these measurements of lipophilic fatty acids used a different set of germline mutants than the other metabolomic comparisons (*mog-3(lf)* instead of *fem-3(gf)* and *fem-3(lf)* instead of *fem-2(lf)*. Bars represent mean ± s.e.m. with two independent biological replicates. **c.** VLCPUFAs with 20-28 carbons were highly enriched in hand-picked samples of N2 and *him-5* males. Longer-chained VLCPUFAs could not be detected due to the snall size of these samples. Bars represent mean ± s.e.m. with two independent biological replicates. **d,** Two VLCPUFAs were detected in worm bodies extracted with methanol, EPA and DPA, which were 4- and 10-fold enriched in old worms over young worms. Bars represent mean ± s.e.m. with six (young adults) and two (day-7 adults) independent biological replicates; *p* values were calculated by unpaired two-sided *t*-tests with Holm-Šídák correction. Source data are provided as a Source Data file.

3. NMR Spectra

(2*O*)- and (3*O*)-acetyl-*S*-methylthioadenosine (acemta#1 4 and acemta#2 5), ^{1}H NMR spectrum (500 MHz, $CDCl_3$)

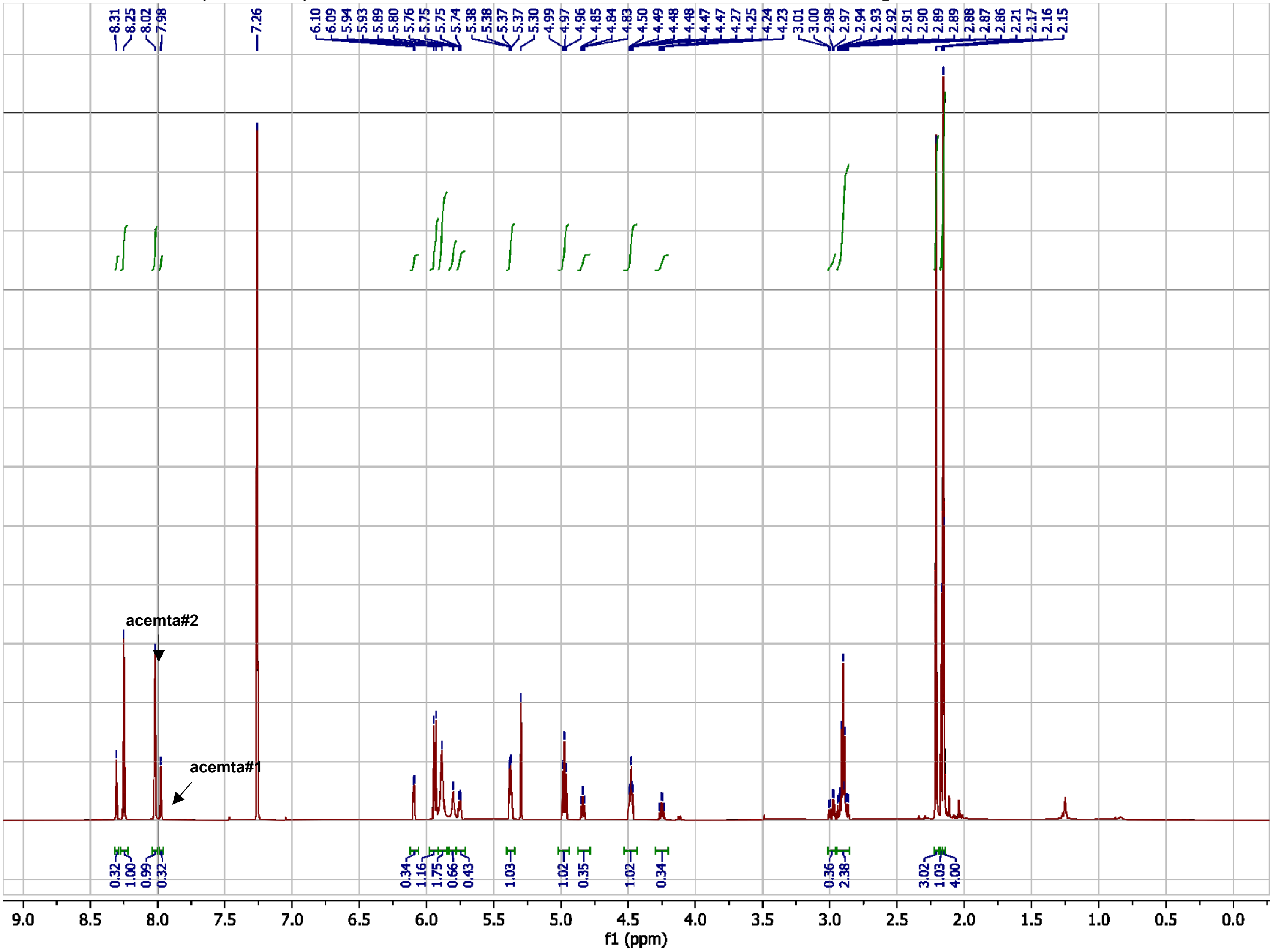

acemta#1 and acemta#2 (4 and 5), ^{13}C NMR spectrum (125 MHz, $CDCl_3$)

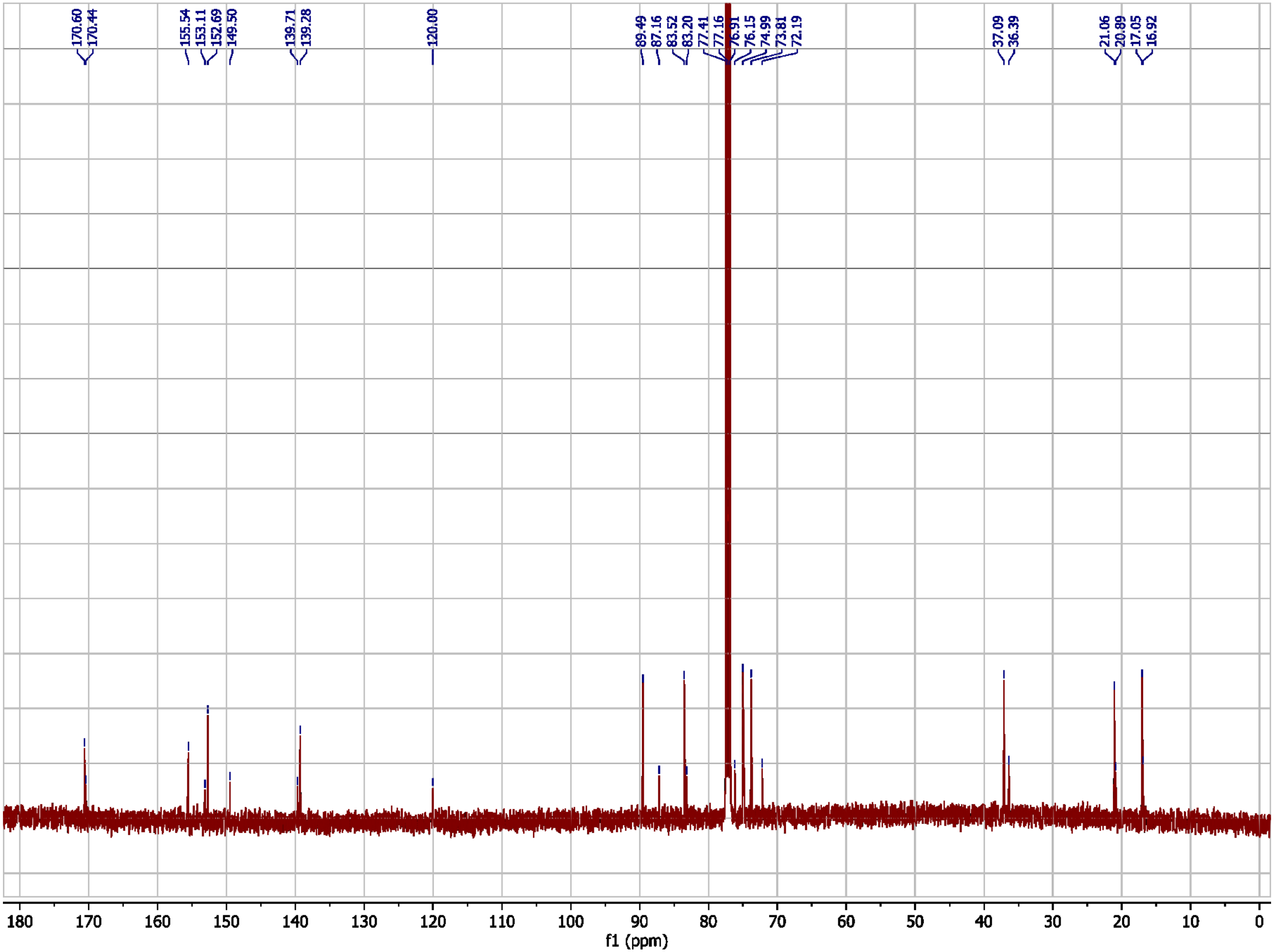

_N,N_-Dimethyltryptophan (24), ^{1}H NMR spectrum (500 MHz, D_6-DMSO)

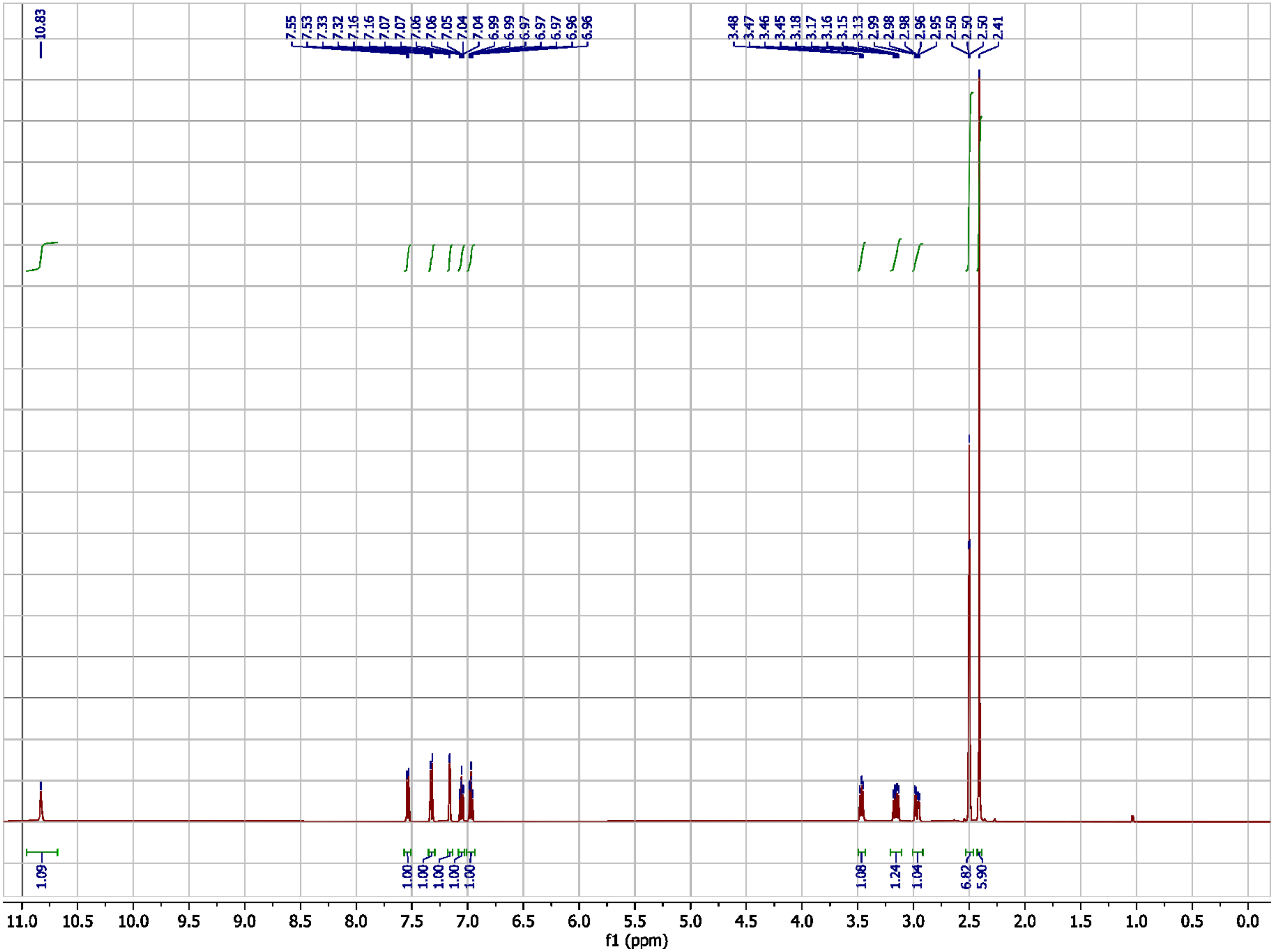

N,N-Dimethyltryptophan (24), ^{13}C NMR spectrum (125 MHz, D_6-DMSO)

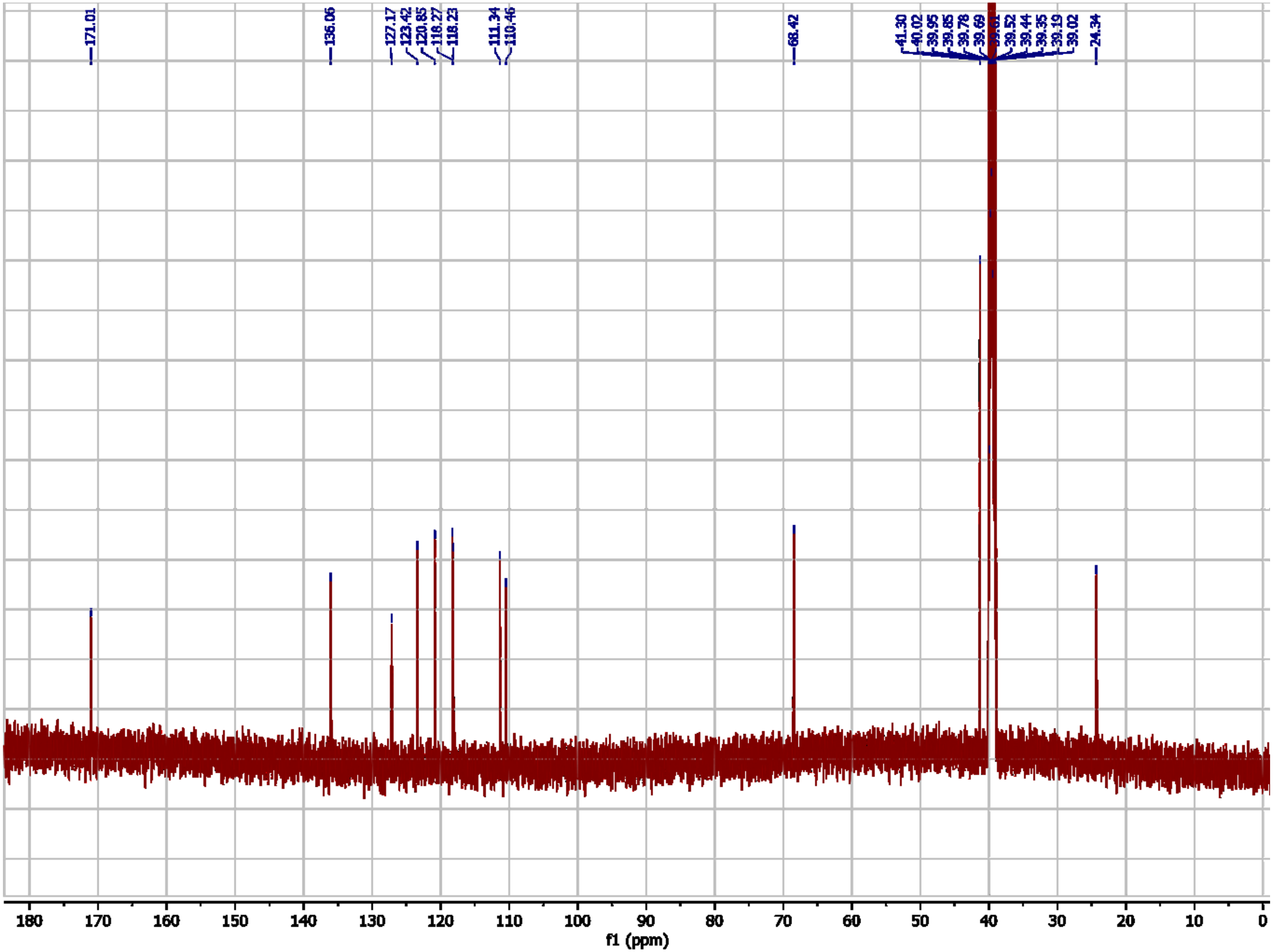

medip#1 (26), ^{1}H NMR spectrum (500 MHz, CD_3OD)

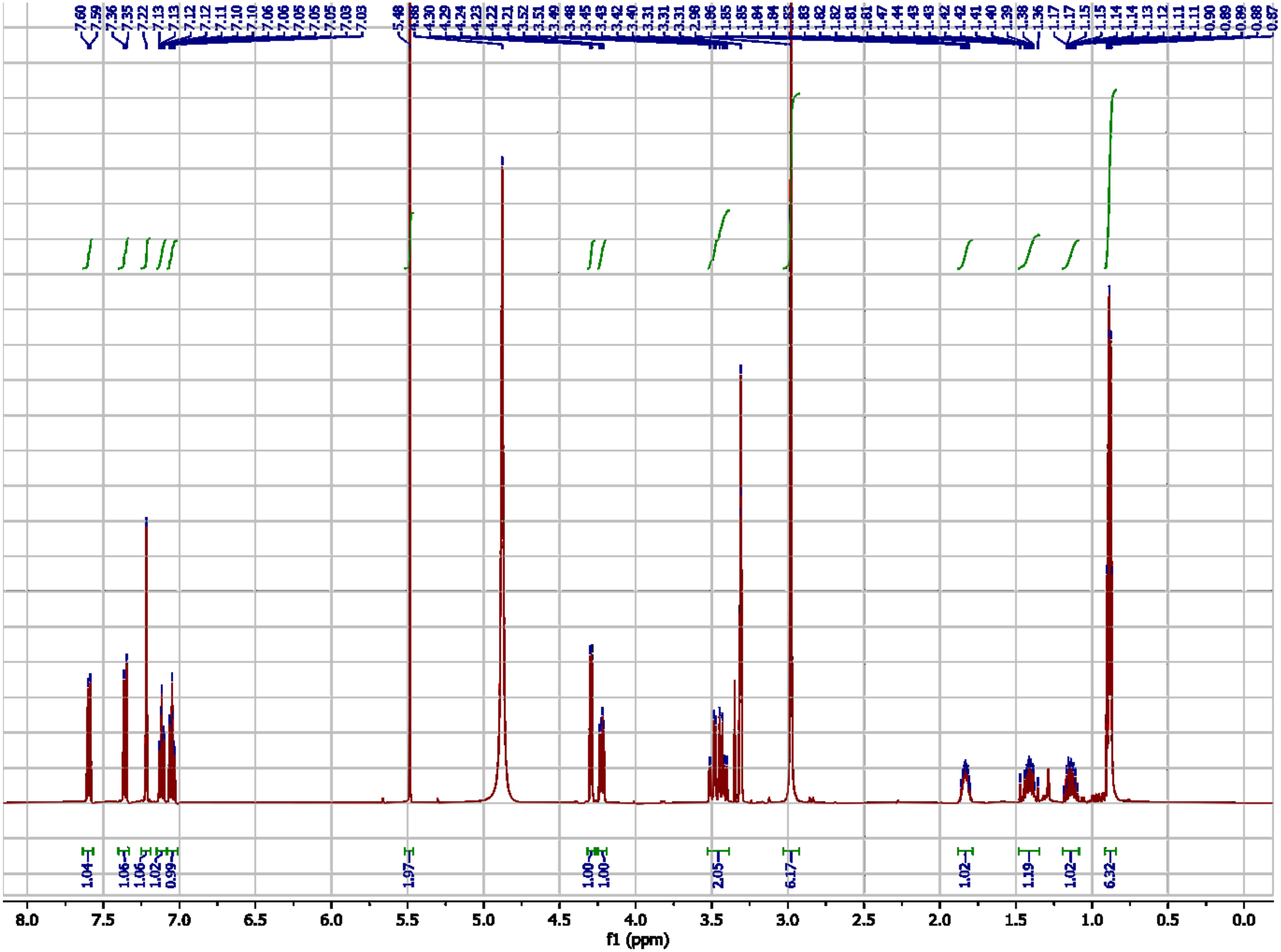

medip#1 (26), ^{13}C NMR spectrum (101 MHz, CD_3OD)

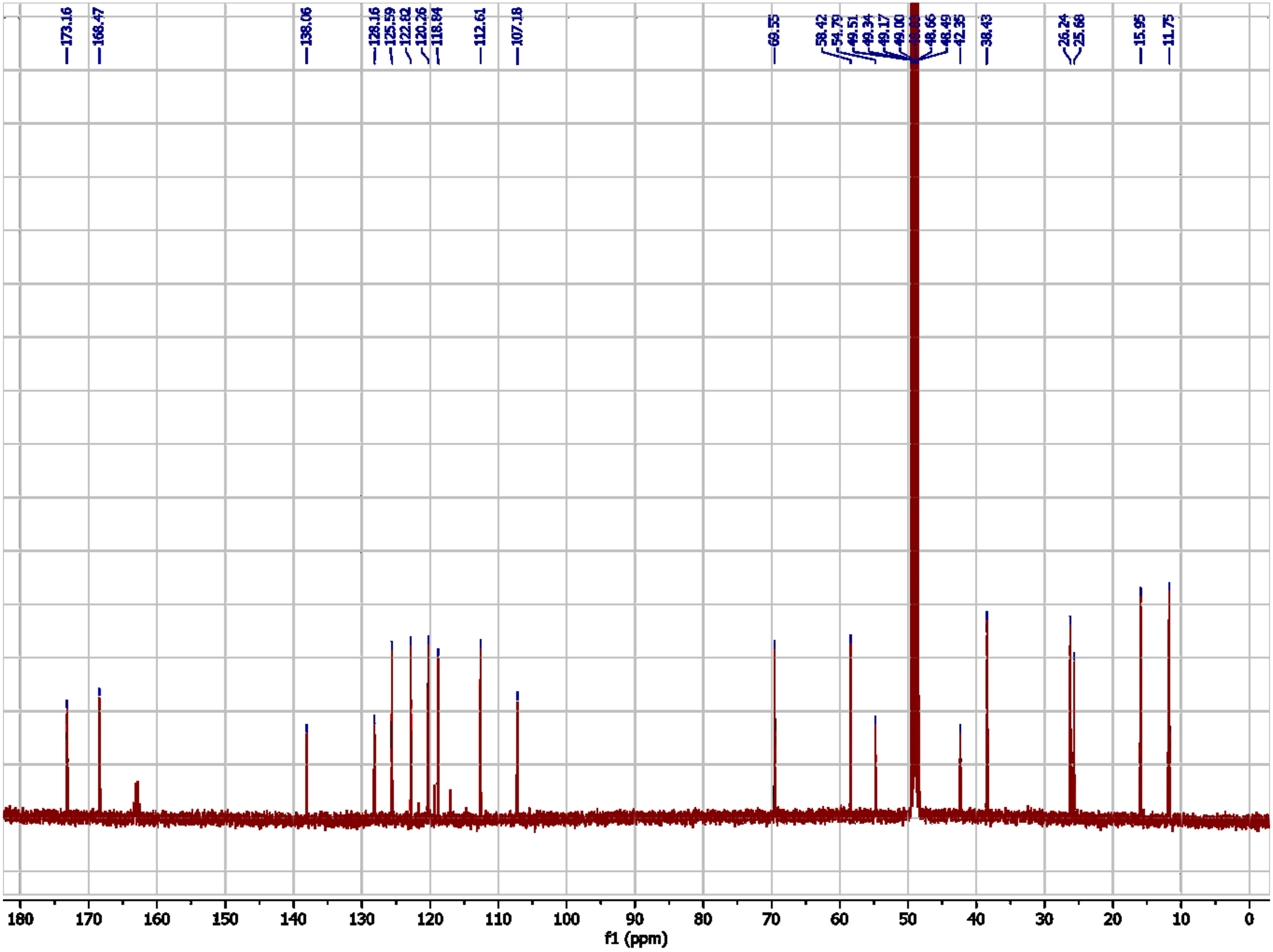

Trp-Ile, ^{1}H NMR spectrum (500 MHz, CD_3OD)

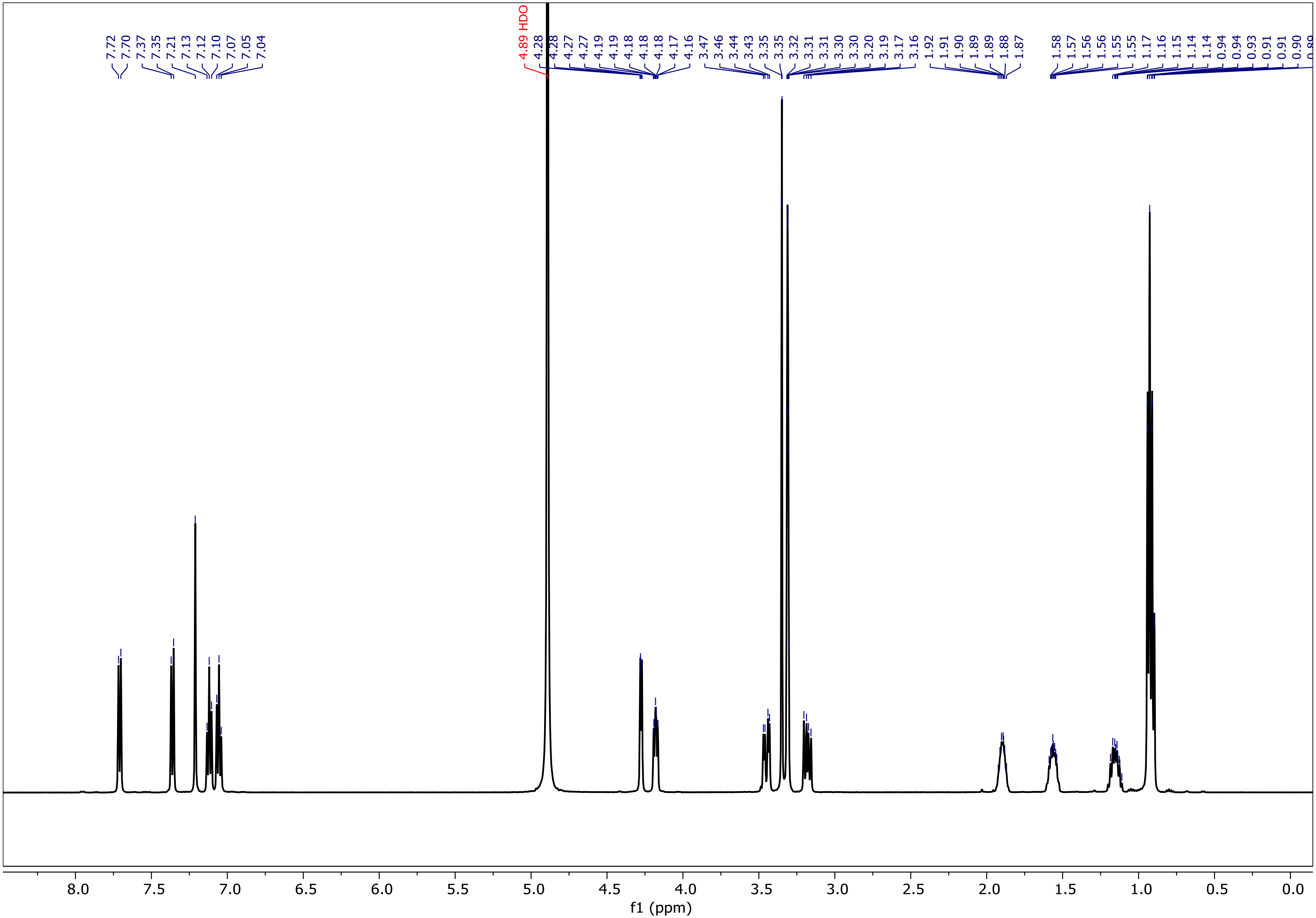

Trp-Ile, ^{13}C NMR spectrum (126 MHz, CD_3OD)

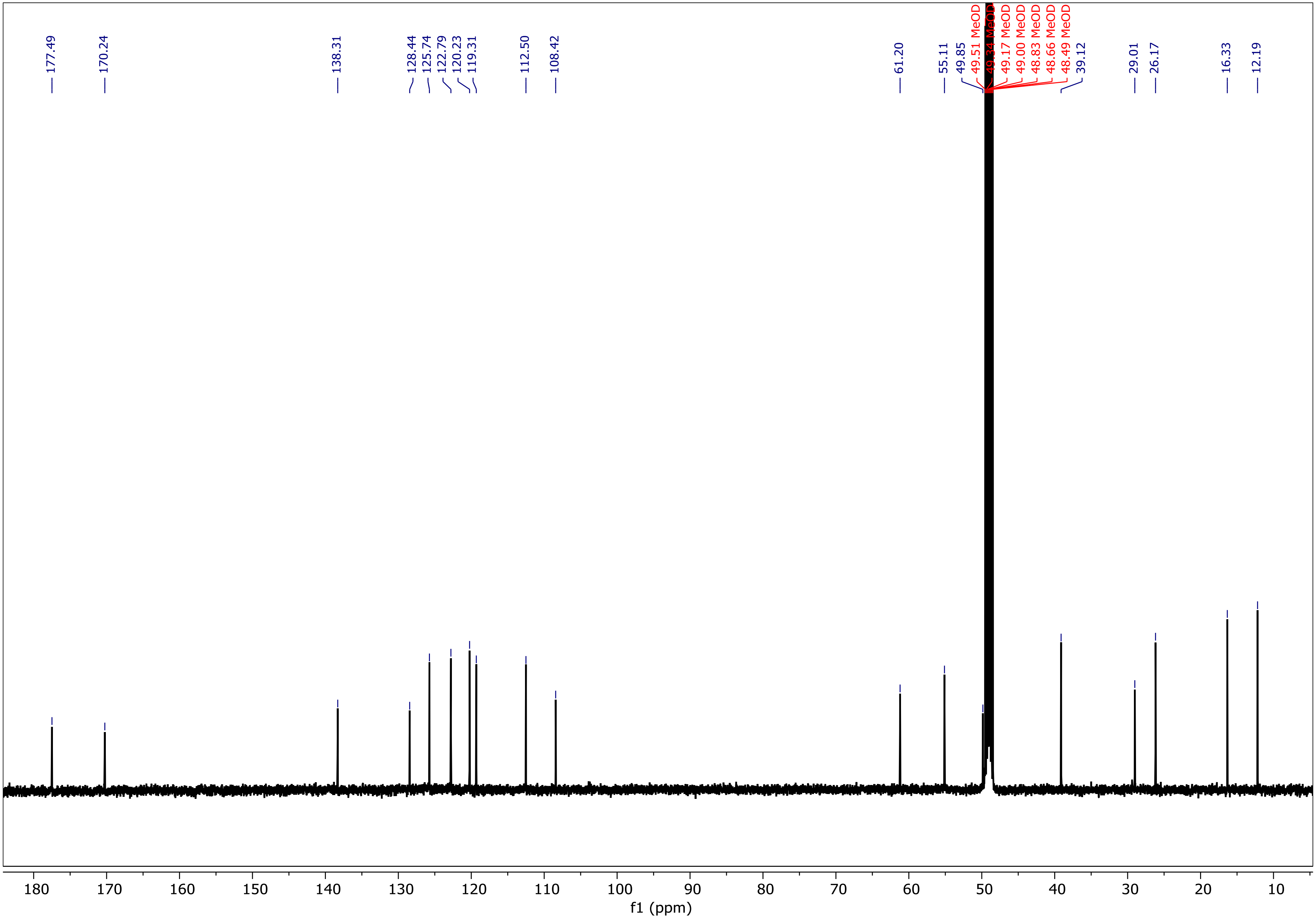

Ile-Trp, ^{1}H NMR spectrum (500 MHz, CD_3OD)

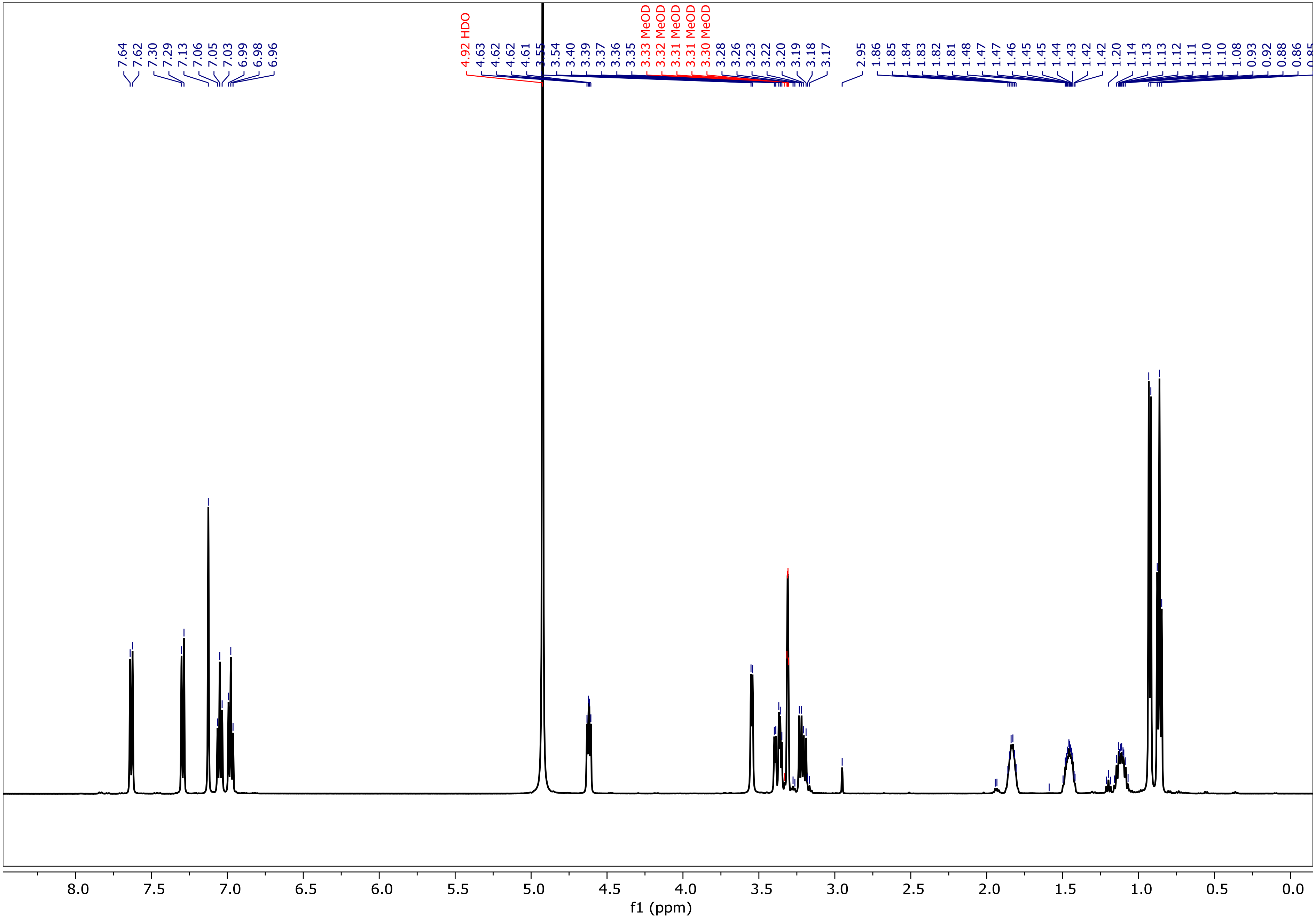

Ile-Trp, ^{13}C NMR spectrum (101 MHz, CD_3OD)

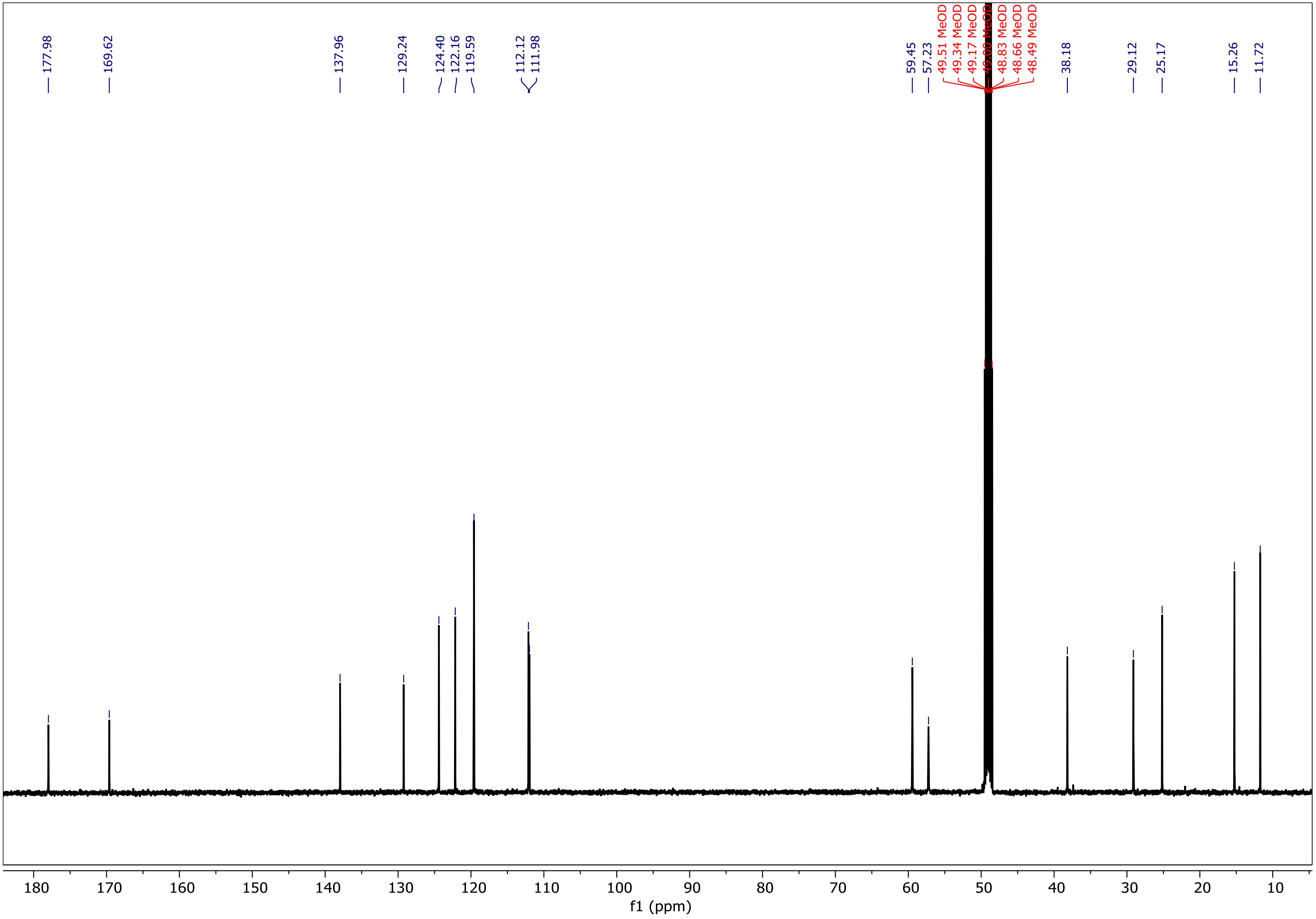

4. Supplementary References

1 Brenner, S. The genetics of Caenorhabditis elegans. *Genetics* **77**, 71-94 (1974).
2 Sulston, J. & Hodgkin, J. *Methods*. (1988).
3 Ludewig, A. H. *et al.* An excreted small molecule promotes C. elegans reproductive development and aging. *Nat Chem Biol* **15**, 838-845, doi:10.1038/s41589-019-0321-7 (2019).
4 Aprison, E. Z. & Ruvinsky, I. Sexually Antagonistic Male Signals Manipulate Germline and Soma of C. elegans Hermaphrodites. *Curr Biol* **26**, 2827-2833, doi:10.1016/j.cub.2016.08.024 (2016).
5 Mok, D. Z., Sternberg, P. W. & Inoue, T. Morphologically defined sub-stages of C. elegans vulval development in the fourth larval stage. *BMC Dev Biol* **15**, 26, doi:10.1186/s12861-015-0076-7 (2015).
6 Aprison, E. Z. & Ruvinsky, I. Balanced trade-offs between alternative strategies shape the response of C. elegans reproduction to chronic heat stress. *PLoS One* **9**, e105513, doi:10.1371/journal.pone.0105513 (2014).
7 MacNeil, Lesley T., Watson, E., Arda, H. E., Zhu, Lihua J. & Walhout, Albertha J. M. Diet-Induced Developmental Acceleration Independent of TOR and Insulin in C. elegans. *Cell* **153**, 240-252, doi:http://dx.doi.org/10.1016/j.cell.2013.02.049 (2013).
8 Frand, A. R., Russel, S. & Ruvkun, G. Functional genomic analysis of C. elegans molting. *PLoS Biol* **3**, e312, doi:10.1371/journal.pbio.0030312 (2005).
9 Brandt, S. D., Moore, S. A., Freeman, S. & Kanu, A. B. Characterization of the synthesis of N,N-dimethyltryptamine by reductive amination using gas chromatography ion trap mass spectrometry. *Drug testing and analysis* **2**, 330-338, doi:10.1002/dta.142 (2010).
10 Hoki, J. S. *et al.* Deep Interrogation of Metabolism Using a Pathway-Targeted Click-Chemistry Approach. *J Am Chem Soc* **142**, 18449-18459, doi:10.1021/jacs.0c06877 (2020).
11 Wrobel, C. J. J. *et al.* Combinatorial Assembly of Modular Glucosides via Carboxylesterases Regulates C. elegans Starvation Survival. *J Am Chem Soc* **143**, 14676-14683, doi:10.1021/jacs.1c05908 (2021).
