# Supplementary material for: Sex-specificity of the C. elegans metabolome
Source: Nat Commun. 2023 Jan 19;14:320. doi: 10.1038/s41467-023-36040-y (PMC9852247; doi:10.1038/s41467-023-36040-y)
Supplement: Supplementary file 3 — Description of Additional Supplementary Files [file 41467_2023_36040_MOESM3_ESM.pdf]

Description of Additional Supplementary Files

File Name: Supplementary Data 1
Description: **List of *him-5*-enriched metabolites.** If the compound was initially detected as enriched in the endo- or exo-metabolome of *him-5* samples it is denoted as a "P" for the pellet/endo-metabolome and/or "S" for the supernatant/exo-metabolome. If a metabolite was not detected in a sample it is denoted "ND". Fold-change values are derived from Metaboseek analysis and those provided for *fem-3* (gf) are relative to WT (N2) samples. If a molecular formula could not be determined from the *m/z* and isotope pattern, the *m/z* for the feature is included in the "Molecular Formula" column.

File Name: Supplementary Data 2
Description: **List of *fem-3* (gf)-enriched compounds.** If the compound was initially detected as enriched in the endo- or exo-metabolome of *fem-3* (gf) samples it is denoted as a "P" for the pellet/endo-metabolome and/or "S" for the supernatant/exo-metabolome. If a metabolite was not detected in a sample, it is denoted "ND". Metabolites not enriched in the metabolome of young (day-1) *him-5* cultures that were enriched in the metabolomes of older *him-5* cultures are denoted with a "*". If a molecular formula could not be determined from the *m/z* and isotope pattern, the *m/z* for the feature is included in the "Molecular Formula" column.

File Name: Supplementary Data 3
Description: **List of metabolites enriched in hand-picked males over hand-picked hermaphrodites.** If a metabolite was not detected in a sample, it is denoted "ND". Fold-change values are derived from Metaboseek analysis and those provided for *fem-3* (gf) are relative to N2 samples. If a molecular formula could not be determined from the *m/z* and isotope pattern, the *m/z* for the feature is included in the "Molecular Formula" column.

File Name: Supplementary Data 4
Description: This file contains a Mass Spectrometric Data Inventory, listing the file names and sample identities for all mass spectrometric data used in this study, which have been deposited at GNPS/MassIVE under accession number ftp://massive.ucsd.edu/MSV000089965/.
