# Supplementary material for: Sex-specificity of the C. elegans metabolome
Source: Nat Commun. 2023 Jan 19;14:320. doi: 10.1038/s41467-023-36040-y (PMC9852247; doi:10.1038/s41467-023-36040-y)
Supplement: Supplementary file 8 — Reporting Summary [file 41467_2023_36040_MOESM8_ESM.pdf]

nature portfolio

Corresponding author(s): Frank C. Schroeder
Ilya Ruvinsky

Last updated by author(s): Jan 5, 2023

Reporting Summary

Nature Portfolio wishes to improve the reproducibility of the work that we publish. This form provides structure for consistency and transparency in reporting. For further information on Nature Portfolio policies, see our Editorial Policies and the Editorial Policy Checklist.

Statistics

For all statistical analyses, confirm that the following items are present in the figure legend, table legend, main text, or Methods section.

n/a	Confirmed	
☐	☒	The exact sample size (*n*) for each experimental group/condition, given as a discrete number and unit of measurement
☐	☒	A statement on whether measurements were taken from distinct samples or whether the same sample was measured repeatedly
☐	☒	The statistical test(s) used AND whether they are one- or two-sided<br>*Only common tests should be described solely by name; describe more complex techniques in the Methods section.*
☐	☒	A description of all covariates tested
☐	☒	A description of any assumptions or corrections, such as tests of normality and adjustment for multiple comparisons
☐	☒	A full description of the statistical parameters including central tendency (e.g. means) or other basic estimates (e.g. regression coefficient) AND variation (e.g. standard deviation) or associated estimates of uncertainty (e.g. confidence intervals)
☐	☒	For null hypothesis testing, the test statistic (e.g. *F*, *t*, *r*) with confidence intervals, effect sizes, degrees of freedom and *P* value noted<br>*Give P values as exact values whenever suitable.*
☒	☐	For Bayesian analysis, information on the choice of priors and Markov chain Monte Carlo settings
☒	☐	For hierarchical and complex designs, identification of the appropriate level for tests and full reporting of outcomes
☒	☐	Estimates of effect sizes (e.g. Cohen's *d*, Pearson's *r*), indicating how they were calculated

Our web collection on statistics for biologists contains articles on many of the points above.

Software and code

Policy information about availability of computer code

Data collection	MS data acquisition was controlled by Chromeleon software (Thermo Scientific v7.3) and Xcalibur software (Thermo Scientific v4.1.31.9).
Data analysis	LC-MS RAW data were converted to mzXML file format using MSConvert (v3.0, ProteoWizard). Data was analyzed using Metaboseek software (version 0.9.7) available here: [https://doi.org/10.5281/zenodo.3360087] and Xcalibur QualBrowser (Thermo Scientific version 4.1.31.9). NMR spectra were processed and baseline corrected using MestreLabs MNOVA software packages version 11.0.0-17609. All statistical analysis were performed with GraphPad Prism (versions 9.2 and 9.4.1) or Metaboseek (version 0.9.7) or R (version 4.1.1).

For manuscripts utilizing custom algorithms or software that are central to the research but not yet described in published literature, software must be made available to editors and reviewers. We strongly encourage code deposition in a community repository (e.g. GitHub). See the Nature Portfolio guidelines for submitting code & software for further information.

Data

Policy information about availability of data

All manuscripts must include a data availability statement. This statement should provide the following information, where applicable:

- Accession codes, unique identifiers, or web links for publicly available datasets
- A description of any restrictions on data availability
- For clinical datasets or third party data, please ensure that the statement adheres to our policy

Source data are provided with this paper. MS and MS/MS data are available at GNPS/MassIVE under accession number ftp://massive.ucsd.edu/MSV000089965/. See MS Data Inventory (attached as a separate Supplementary Data file) for file names and sample identities.

Human research participants

Policy information about studies involving human research participants and Sex and Gender in Research.

Reporting on sex and gender	NA
Population characteristics	NA
Recruitment	NA
Ethics oversight	NA

Note that full information on the approval of the study protocol must also be provided in the manuscript.

Field-specific reporting

Please select the one below that is the best fit for your research. If you are not sure, read the appropriate sections before making your selection.

☒ Life sciences ☐ Behavioural & social sciences ☐ Ecological, evolutionary & environmental sciences

For a reference copy of the document with all sections, see nature.com/documents/nr-reporting-summary-flat.pdf

Life sciences study design

All studies must disclose on these points even when the disclosure is negative.

Sample size	Sample sizes were chosen based on previous experience for each experiment, specifically for metabolomics experiments we based our choices on Artykhin et al., JACS 2018 (https://pubs.acs.org/doi/full/10.1021/jacs.7b11811), and for the biological experiments on Ludewig et al., Nat. Chem. Biol. 2019 (https://pubmed.ncbi.nlm.nih.gov/31320757/). No statistical methods were used to predetermine sample size.
Data exclusions	No data were arbitrarily excluded.
Replication	Experiments were usually performed in several independent replicates (often three or more) as indicated, at different times. Several experiments were replicated in two different laboratories. All attempts at replication were successful.
Randomization	Age synchronized populations of animals were produced and assigned randomly to experimental or control groups.
Blinding	All mass spectrometric data acquisition was performed blinded. During extraction and sample processing for metabolomics the identities of samples were unknown to the researchers. Likewise, lifespan and developmental pace experiments in which different compounds were compared were performed blinded. Assessment of differences between control and treated animals relied on objective, predetermined criteria, also in cases where blinding was not practical, e.g., since sex mutants have different properties or have obvious shape or size differences. .

Reporting for specific materials, systems and methods

We require information from authors about some types of materials, experimental systems and methods used in many studies. Here, indicate whether each material, system or method listed is relevant to your study. If you are not sure if a list item applies to your research, read the appropriate section before selecting a response.

Materials & experimental systems

n/a	Involved in the study
☒	☐ Antibodies
☒	☐ Eukaryotic cell lines
☒	☐ Palaeontology and archaeology
☐	☒ Animals and other organisms
☒	☐ Clinical data
☒	☐ Dual use research of concern

Methods

n/a	Involved in the study
☒	☐ ChIP-seq
☒	☐ Flow cytometry
☒	☐ MRI-based neuroimaging

Animals and other research organisms

Policy information about studies involving animals; ARRIVE guidelines recommended for reporting animal research, and Sex and Gender in Research

Laboratory animals	Strain, sex, and age information for C. elegans used in this study is provided in the figure legends, methods, and supplementary information files. The following strains were used in this study: wildtype Bristol N2, CB4088 him-5(e1490), DH245 fem-2(b245), JK816 fem-3(q20) (gain-of function), CB3844 fem-3(e2006) (loss-of-function), SS104 glp-4(bn2), CB4037 glp-1(e2141), JK569 mog-3(q74), PS7922 cest-5.1(syb1131), FCS51 cest-5.1(syb1131);him-5(e1490), PHX3933 cest-5.1(syb1131);cest-5.2(syb3933), GR1395 mgIs49 [mlt-10::GFP-pest;ttx-1::GFP], UR936 fsEx445[Pnhx-2::FEM-3(+)::SL2::mCherry::unc-54_3'UTR;Psulp-3::gfp].
Wild animals	No wild animals were used in this study.
Reporting on sex	The paper is explicitly about differences between sexes.
Field-collected samples	No field-collected samples were used in this study.
Ethics oversight	No ethical approval was required

Note that full information on the approval of the study protocol must also be provided in the manuscript.
